# Inhibition of CAV1 attenuates diabetic cardiomyopathy through reducing ferroptosis via activating NRF2/GCLC signaling pathway

**DOI:** 10.7150/thno.107367

**Published:** 2025-03-31

**Authors:** Guangru Li, Ruiqing Liu, Zeyan Peng, Shengzheng Zhang, Runjia Sun, Ziwei Wang, Jing Li, Yang Gao, Yang Xu, Jianlin Cui, Jie Liu, Jie Yan, Lei Cao, Shan Ren, Yushun Chu, Lifeng Feng, Liang Yang, Yanna Shen, Zhi Qi

**Affiliations:** 1 Department of Molecular Pharmacology, School of Medicine, Nankai University; Department of Cardiology, Beichen Hospital, Nankai University, Tianjin, 300071, China.; 2 School of Medical Technology, Tianjin Medical University, Tianjin, 300203, China.; 3 The Third Central Hospital of Tianjin, 83 Jintang Road, Hedong District, Tianjin, 300170, China.; 4 Tianjin Key Laboratory of Extracorporeal Life Support for Critical Diseases.; 5 Artificial Cell Engineering Technology Research Center, Tianjin, China.; 6 Tianjin Institute of Hepatobiliary Disease, Tianjin, China.; 7 National Key Laboratory of Kidney Diseases, Chinese PLA General Hospital, Beijing, China.; 8 Tianjin Key Laboratory of General Surgery in Construction, Tianjin Union Medical Center, Tianjin, 300000, China.; 9 The First Department of Critical Care Medicine, The First Affiliated Hospital of Shihezi University, Shihezi, 832003, China.; 10 Key Laboratory of Bioactive Materials, Ministry of Education, Nankai University, Tianjin, 300071, China.

**Keywords:** Diabetic cardiomyopathy, Caveolin-1, NRF2, GCLC, Ferroptosis

## Abstract

**Background**: Diabetic cardiomyopathy (DCM), a prevalent complication of diabetes, is a major cause of heart failure and death among patients with diabetes. However, the pathological mechanisms underlying the development of DCM remain unclear. This study aims to investigate the role and underlying mechanisms of caveolin-1 (CAV1) in DCM.

**Methods**: DCM model was established *in vivo* through intraperitoneal injection of streptozotocin in mice and *in vitro* through high-glucose (HG) treatment in neonatal rat ventricular myocytes (NRVMs). CAV1-knockout (CAV1-KO) and overexpression (by injecting adeno-associated virus 9 (AAV9) encoding CAV1) mice were utilized to explore the role of CAV1 in DCM. Nuclear factor erythroid 2-related factor 2 (NRF2)-KO and AAV9-NRF2 mice and ML385 (an NRF2 inhibitor) were used to investigate the effect of NRF2 on DCM.

**Results**: CAV1 expression was significantly increased in the cardiac tissues of diabetic mice and HG-treated NRVMs. CAV1 deficiency significantly alleviated diabetes-induced myocardial hypertrophy, fibrosis, abnormal mitochondria, excessive reactive oxygen species production, and ferroptosis. Conversely, cardiac-specific overexpression of CAV1 exacerbated cardiac dysfunction and myocardial histological abnormalities caused by diabetes. Mechanistically, CAV1 directly bound to NRF2 and inhibited its nuclear translocation, reducing the transcription of glutamate cysteine ligase catalytic subunit (GCLC), accumulating excess peroxide, and inducing ferroptosis and myocardial injury.

**Conclusion**: CAV1 exacerbates the progression of DCM by suppressing the NRF2/GCLC pathway, suggesting that targeting CAV1 is a potential therapeutic approach for DCM.

## Introduction

The prevalence of diabetes mellitus (DM) is increasing worldwide. Diabetic cardiomyopathy (DCM), a severe complication of diabetes, is a major cause of mortality and morbidity among diabetic patients 1, 2. DCM is characterized by myocardial fibrosis, hypertrophy, oxidative stress, cardiomyocyte death, and cardiac dysfunction, causing a high risk of heart failure syndrome 3, 4. The pathological mechanisms of DCM remain partially elucidated, and effective targets for preventing or mitigating the progression of DCM are urgently required.

Caveolin-1 (CAV1) is the major structural protein of caveolae and comprises four domains (Figure S1A), among which the caveolin-scaffolding domain (CSD) is a highly conserved region that mediates direct protein-protein interactions between CAV1 and various signaling molecules 5. Previous studies have suggested that aberrant CAV1 expression is associated with multiple diseases, including atherosclerosis 6, pulmonary hypertension 7, and cancer 8. CAV1-knockout protects against the progression of atherosclerosis by increasing endothelial autophagy and attenuating vascular inflammation 6. CAV1 deletion attenuates angiotensin II-induced hypertensive vascular remodeling and inflammation 9. Hypoxia-induced upregulation of CAV1 in hepatocellular carcinoma drives tumorigenesis and metastasis 10. Rosuvastatin improves heart rate and blood pressure variabilities by decreasing CAV1 expression and promoting NOS function in ApoE-KO induced dyslipidemic mice 11. However, the role of CAV1 in DCM remains controversial. Hu et al. demonstrated that CAV1 interacts directly with succinate dehydrogenase subunit A, promoting its ubiquitination and proteasomal degradation, causing mitochondrial dysfunction and mitochondria-derived apoptosis in DCM; however, silencing CAV1 reduces apoptosis and improves mitochondrial function 12. In contrast, Gong et al. reported that CAV1 knockdown promotes diabetes-induced cardiac injury by activating NF-κB-mediated inflammatory signaling 13. Thus, the molecular mechanism by which CAV1 regulates DCM requires further investigation.

Ferroptosis is an iron-dependent, lipid peroxide-driven form of cell death owing to a redox imbalance caused by reactive oxygen species (ROS) overproduction or antioxidant system dysfunction 14, 15. Ferroptosis has been identified in the pathological processes of many cardiovascular diseases, including atherosclerosis 16, myocardial ischemia-reperfusion injury 17, and DCM 18. Wang et al. highlighted the pivotal role of ferroptosis in DCM, demonstrating that inhibition of ferroptosis by liproxstatin-1 prevents the development of cardiac dysfunction 18. Similarly, Tang et al. reported that irisin mitigates cardiac remodeling and dysfunction by suppressing ferroptosis in STZ-induced type 1 diabetes mellitus mice 19. These results highlight the importance of ferroptosis in DCM. The antioxidant glutathione (GSH) directly influences the activity of glutathione peroxidase-4 (GPX4), a powerful scavenger of lipid peroxidation 20, 21. Thus, impaired GSH metabolism is a major trigger for ferroptosis. The glutamate cysteine ligase (GCL) is the rate-limiting enzyme in the biosynthesis of GSH, with its catalytic subunit GCLC being pivotal for the synthesis of γ-glutamyl cysteine from glutamate and cysteine 22, 23. Nuclear factor erythroid 2-related factor 2 (NRF2) is a master regulator of cellular redox homeostasis through transcriptional regulation of antioxidant enzymes 24, 25. Research has indicated that the activation of NRF2 protects against DCM by inhibiting ferroptosis 18. However, the regulatory mechanisms of NRF2 on ferroptosis in DCM is still limited. Whether NRF2 alleviates DCM by regulating GCLC to inhibit ferroptosis has not yet been reported.

Syringaresinol (SYR) is a natural polyphenolic compound with antioxidant and anti-inflammatory properties 26, 27. Studies have showed that SYR significantly attenuates oxidative stress-induced skin aging by regulating autophagy 28. Moreover, SYR improves sepsis-induced cardiac dysfunction by inhibiting inflammation and pyroptosis 29. Our previous study indicated that SYR protects against DCM by alleviating inflammatory responses and oxidative stress 30.

In this study, we demonstrate that CAV1 is upregulated in DCM. CAV1 deficiency significantly improves diabetes-induced myocardial injury and cardiac dysfunction. Conversely, cardiac-specific CAV1 overexpression exacerbates cardiac hypertrophy and fibrosis, and further deteriorates cardiac dysfunction in diabetic mice. Mechanistically, CAV1 inhibits the nuclear translocation of NRF2 by interacting directly with NRF2, suppressing GCLC transcription, causing ferroptosis of cardiomyocytes. These findings collectively suggest that CAV1 may serve as a promising therapeutic target for DCM.

## Materials and methods

### Animal experiments

All animal care and experimental procedures were approved by the Institutional Research Ethics Committee of Nankai University (2024-SYDWLL-000643) and strictly conformed to the Guide for the Care and Use of Laboratory Animals of Nankai University. Wildtype male C57BL/6 J mice aged 6-8 weeks and neonatal rats were purchased from SPF Biotechnology Co., Ltd. (Beijing, China). CAV1-knockout (CAV1-KO) and NRF2-knockout (NRF2-KO) mice on C57BL/6 J background were purchased from Shanghai Model Organisms Center, Inc. (Shanghai, China). CAV1 genotyping was conducted by PCR using the primers listed in Table S1. For CAV1 knockout homozygotes, the PCR product of P1 and P2 was 681 bp fragment, while no band was observed for P3 and P4. For wild type, P1 and P2 was 1186 bp fragment, P3 and P4 produced 658 bp fragment. Genotyping PCR was performed to identify NRF2 using the primers listed in Table S2. For NRF2 knockout homozygotes, the PCR result of P1 and P2 was 1467 bp fragment, P3 and P4 did not yield any bands. For wild type, the PCR of P1 and P2 produced 5727 bp fragment, P3 and P4 yielded 489 bp fragment. The PCR reaction reagents from Takara (Beijing, China). The primers were synthesized by Sangon Biotech Co., Ltd.

Type 1 diabetes mellitus was induced by administering streptozotocin (STZ, 50 mg/kg body weight) via intraperitoneal injection for 5 consecutive days. After a week, mice with fasting blood glucose above 11.1 mmol/L were considered as diabetic and were included in the study. To construct cardiomyocyte-specific overexpression of CAV1 or NRF2 mice, recombinant adeno-associated virus serotype 9 (AAV9) vectors containing the cardiac troponin T promoter to drive the expression of CAV1 (AAV9-CAV1) or NRF2 (AAV9-NRF2), as well as negative control (AAV9-Vector), were purchased from Obio Technology Co., Ltd. (Shanghai, China). Wild-type diabetic mice and healthy mice were injected via the tail vein with AAV9-CAV1, AAV9-NRF2, or AAV9-vector (2×10^11^ v.g./mouse). To determine whether the beneficial effect of CAV1-KO on DCM depends on NRF2. CAV1-KO mice received intraperitoneal injection of ML385 (an NRF2-specific inhibitor, 30 mg/kg; MCE, Shanghai, China) after establishing the diabetic model, according to previous studies 31, 32. Regarding the treatment with SYR, wild-type and AAV9-CAV1 diabetic mice were administered SYR (25 mg/kg body weight) by oral gavage every other day after establishing the diabetic model, according to previous study 30. Blood glucose levels and body weights of the mice were monitored weekly. After 8 weeks of establishing the diabetic model, mice were euthanized by cervical dislocation under anesthesia.

### Echocardiography

Cardiac function was evaluated using transthoracic echocardiography (vevo2100, Visualsonics, USA) at the 8 weeks after establishing the diabetic model. Mice were lightly anesthetized with isoflurane and placed in the supine position. M-mode tracing was performed through the short axis of the left ventricle. Left ventricular fractional shortening (FS) and ejection fraction (EF) were calculated for at least four consecutive cardiac cycles.

### Cell culture

Neonatal rat ventricular myocytes (NRVMs) were isolated from 1- to 2-day-old Sprague Dawley rats, as described previously 33. The cellular model of diabetes-induced myocardial injury was established by treating NRVMs with 33 mM glucose. Lentiviral vector encoding rat CAV1 (OE-CAV1), rat NRF2 (OE-NRF2) and control vector were constructed by MiaoLing Biology Co., Ltd. (Wuhan, China). NRVMs were infected with lentivirus in the presence of 8 μg/mL polybrene for 8 hours. Small interfering RNA targeting CAV1 (si-CAV1), NRF2 (si-NRF2), GCLC (si-GCLC) and non-targeting siRNA (NC) were purchased from Shanghai GenePharma Co., Ltd. (Shanghai, China), and transfected with Hieff Trans® Liposomal Transfection Reagent (Yeasen, Shanghai, China), according to the manufacturer's instruction. Ferroptosis agonist erastin (5 μM, MCE, Shanghai, China) and inhibitor ferrostatin-1 (10 μM, MCE, Shanghai, China) were used to investigate the role of ferroptosis in DCM, according to previous studies 18, 34.

### Histological analysis

For histopathological analysis, freshly harvested cardiac tissues were fixed in 4% paraformaldehyde solution, embedded in paraffin, and serially sectioned at 5-µm thickness. The cross-sectional area of cardiomyocytes was detected using wheat germ agglutinin (WGA, Sigma, MO, USA) staining. Cardiac fibrosis was assessed using the Masson's trichrome Staining Kit (Solarbio, Beijing, China). Images were captured with a microscope (Olympus, BX53) and analyzed using the Image J software.

For immunohistochemistry, the tissue sections were deparaffinized, antigen-repaired with citrate buffer, blocked with 5% goat serum albumin, and incubated with CAV1 antibody (Santa Cruz, CA, USA) or NRF2 antibody (Sigma, MO, USA) overnight at 4 °C, followed by incubation with horseradish peroxidase (HRP)-conjugated secondary antibody (ZSGB-BIO, Beijing, China) at room temperature for 1 hour. Chromogenic revelation was performed with the DAB kit (ZSGB-BIO, Beijing, China). Images were captured using a microscope (Olympus, IX71).

### Detection of myocardial enzymes, GSH, MDA and Fe^2+^

Serum creatine kinase isoenzyme (CK-MB) and lactate dehydrogenase (LDH) were detected using an automatic biochemical analyzer (Beckman Coulter, CA, USA). Cardiac GSH levels were assessed with the Glutathione ELISA kit (Elabscience, Wuhan, China), according to the manufacturer's instructions. Intracellular GSH was detected using the Glutathione ELISA kit (Elabscience, Wuhan, China), according to the manufacturer's instructions. Cardiac malondialdehyde (MDA) levels were detected following the manufacturer's protocol of the MDA assay kit (Beyotime, Shanghai, China). Intracellular ferrous ions were evaluated by using the Cell Ferrous Iron Colorimetric Assay kit (Elabscience, Wuhan, China), according to the manufacturer's instructions.

### Western Blotting

Total protein was extracted from heart tissues and NRVMs using RIPA buffer (Solarbio, Beijing, China) containing protease and phosphatase inhibitors. Proteins were collected through centrifugation at 12,000 g at 4 °C for 30 minutes. Nuclear and cytoplasmic proteins were extracted from NRVMs using the Nuclear and Cytoplasmic Protein Extraction Kit (Beyotime, Shanghai, China). Protein samples were separated by 10-12% SDS-PAGE and transferred to PVDF membranes. The membranes were blocked with 5% bovine serum albumin and incubated at 4 °C overnight with the following primary antibodies: anti-CAV1 (CST, MA, USA), anti-NRF2 (Sigma, MO, USA), anti-GCLC (ABclonal, Wuhan, China), anti-GPX4 (Abcam, MA, USA), anti-Tubulin (ABclonal, Wuhan, China) and anti-Histone H3 (CST, MA, USA). Subsequently, the membranes were incubated with HRP-conjugated secondary antibody for 1 hour at room temperature. Protein bands were visualized using ECL reagent (Shandong Sparkjade Biotechnology Co., Ltd.) and quantified using the Image J software.

### Quantitative real-time PCR

Total RNA was extracted from heart tissues and NRVMs using the Trizol reagent (Solarbio, Beijing, China) according to the manufacturer's protocol. First-strand cDNA synthesis from 1 μg of total RNA was performed using the EasyScript All-in-One First-Strand cDNA Synthesis SuperMix (TransGen Biotech, Beijing, China). Quantitative real-time PCR was performed using the PerfectStart Green qPCR SuperMix (TransGen Biotech, Beijing, China) in a Roche LightCycler 96 system. The primer sequences are listed in Table S3.

### Immunofluorescence staining

NRVMs cultured in confocal dishes were fixed with 4% paraformaldehyde for 20 minutes and permeabilized with 0.1% Triton X-100 for 15 minutes. After blocking in 1% BSA for 1 hour, NRVMs were incubated with primary antibodies against CAV1 (Santa Cruz, CA, USA) and NRF2 (Sigma, MO, USA) overnight at 4 °C. Subsequently, NRVMs were incubated with Alexa Fluo-488/594 antibody (ZSGB-BIO, Beijing, China) at room temperature for 1 hour and counter-stained with DAPI for 5 minutes. Images were acquired using a confocal microscope (Olympus, FV1000) and analyzed using the Image J software.

### Dihydroethidium (DHE) and lipid peroxides staining

Briefly, the fresh heart tissues were embedded in OCT solution and sectioned at 6 µm thickness. The frozen sections and NRVMs were washed thrice with PBS, followed by DHE staining (Solarbio, Beijing, China) for 30 minutes. Images were acquired using a fluorescence microscope (Olympus, BX53, IX71) and quantified using the Image J software.

To detect the accumulation of lipid peroxides in NRVMs, the Liperfluo-Cell Lipid Peroxidation Assay Kit (Dojindo, Kumamoto, Japan) was conducted following the manufacturer's instructions. Fluorescent images were captured using a confocal microscope (Olympus, FV1000).

### Calcein-AM/PI staining

NRVMs were analyzed using the Calcein-AM/PI cell viability/cytotoxicity assay kit (Beyotime, Shanghai, China) according to the manufacturer's instructions. Briefly, NRVMs were incubated with Calcein-AM/PI working solution at 37 °C for 30 minutes. Images were acquired using a fluorescence microscope (Olympus, IX71). The percentage of PI-positive cells were quantified.

### Co-IP analysis

NRVMs were harvested and lysed in RIPA buffer (Solarbio, Beijing, China) containing phosphatase and protease inhibitors. The cell lysates were incubated with 2 μg of indicated antibodies against CAV1 (CST, MA, USA) or control IgG (ABclonal, Wuhan, China) overnight at 4 °C. Subsequently, 50 µL of protein A/G agarose (Santa Cruz, CA, USA) were added and incubated for 6 hours at 4 °C under rotation. The bound proteins were eluted in 2× SDS sample buffer and further analyzed using western blotting.

### GST-pull down

Bacteria-expressed GST or GST-CAV1 proteins were immobilized on glutathione-sepharose 4B beads (GenScript, Nanjing, China) and washed with GST binding/wash buffer (Sangon Biotech, Shanghai, China). Subsequently, these beads were incubated with HEK293T cell lysates expressing FLAG-NRF2 overnight at 4 °C under rotation. After washing these beads with GST binding/washing buffer, the bound proteins were eluted with 2×SDS sample buffer and subjected to western blotting.

### Mitochondrial assay

Mitochondrial morphology in mouse heart was observed using transmission electron microscopy (TEM). Myocardial samples (2 mm^3^) were quickly isolated and immediately fixed in cold 2.5% glutaraldehyde (Solarbio, Beijing, China). Post-fixation, embedding, and sectioning of the samples were performed by Hangzhou Yanqu Information Technolog Co., Ltd (Hangzhou, China). TEM images were obtained using a transmission electron microscope (Hitachi HT7700 Exalens).

For analysis of mitochondrial morphology in NRVMs, NRVMs incubated in confocal dishes were stained with MitoTracker (MCE, Shanghai, China) for 30 minutes. Nuclei were counter-stained with Hoechst 33342. Fluorescent signals were captured using a confocal microscope (Olympus, FV1000).

### Dual-luciferase reporter assay

The GCLC (mouse) firefly luciferase reporter plasmid PGL4.10-GCLC-luc2 and PGL4.71-renilla luciferase plasmid were designed and synthesized by Hanbio Biotechnology Co., Ltd. (Shanghai, China). HEK293T cells were cultured to approximately 80% confluence and transfected with PGL4.71 and PGL4.10-GCLC-luc2 plasmids with or without NRF2 plasmid. 48 hours after transfection, the luciferase reporter activity was measured using the Dual-Luciferase Reporter Gene Assay kit (Yeasen, Shanghai, China) according to the manufacturer's instructions. Firefly luciferase values were normalized to renilla luciferase values.

### Statistical analyses

Statistical analyses were conducted with GraphPad Prism software. Data were presented as mean ± SD. Differences between two groups were analyzed using the unpaired Student's t-test. For multiple groups, one-or two-way ANOVA was performed, followed by Tukey's multiple comparisons test. Statistical significance was defined as *P* < 0.05.

## Results

### CAV1 expression is increased in DCM

**Figure 1:**
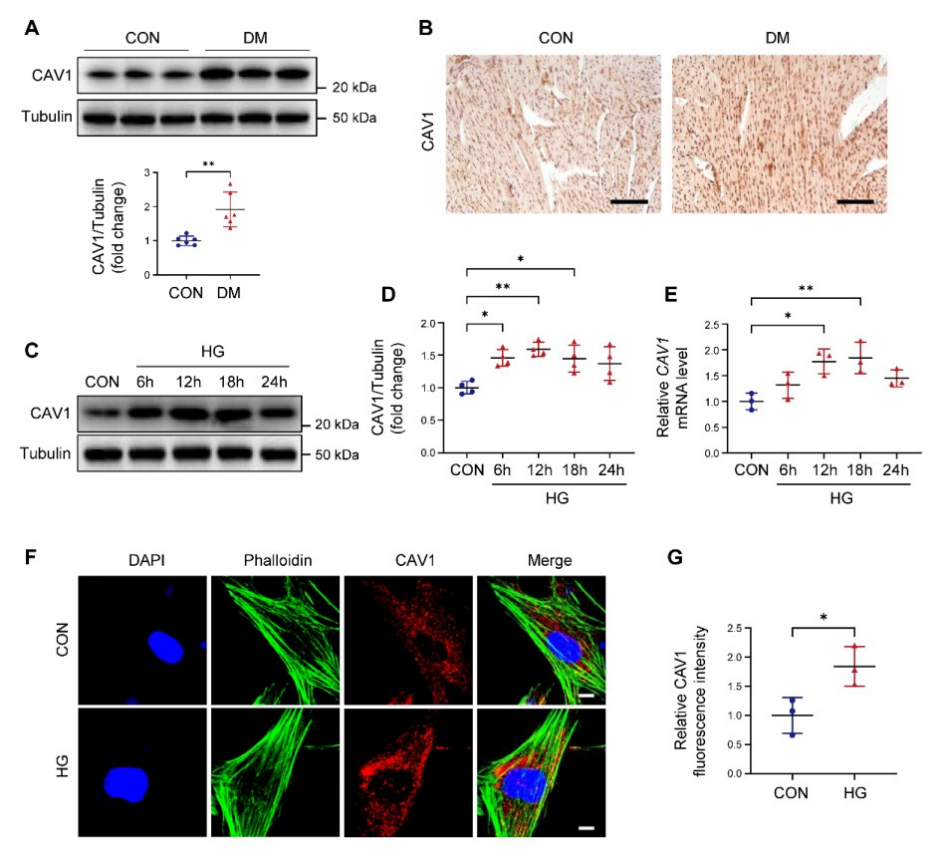
Expression of caveolin-1 (CAV1) is increased in the heart of diabetic mice and high glucose (HG)-treated neonatal rat ventricular myocytes (NRVMs). (**A**) Representative western blotting and quantification of CAV1 expression in cardiac tissues of healthy and diabetic mice. (**B**) Immunohistochemistry for CAV1 in heart sections. Scale bar = 100 μm. (**C-D**) NRVMs were treated with HG (33 mM) for 6, 12, 18, and 24 hours. Representative immunoblotting images and quantitative analysis of CAV1. (**E**) qRT-PCR analysis of *CAV1* mRNA expression. (**F-G**) Representative fluorescence images of CAV1 (red), Phalloidin (green), and DAPI (blue) in NRVMs treated with HG for 12 hours. Scale bar = 5 μm. Data were expressed as mean ± SD. ^*^P < 0.05, ^**^P < 0.01.

The expression of CAV1 in DCM was first determined. Our data indicated that CAV1 protein levels were dramatically increased in hearts of diabetic mice compared with control mice (Figure 1A). Similarly, immunohistochemistry revealed a higher intensity of CAV1 in the cardiac tissue of diabetic mice than in the control group (Figure 1B), suggesting that DCM was associated with a significant increase of CAV1. Subsequently, we established an *in vitro* DCM model induced by high glucose (HG) in NRVMs. Upon exposure to HG, the protein and mRNA levels of CAV1 were remarkably upregulated in NRVMs (Figure 1C-E). Meanwhile, we studied the effect of mannitol, a hypertonic control, on the expression of CAV1 in NRVMs. As anticipated, mannitol treatment had no impact the mRNA expression of *CAV1* (Figure S1B). Moreover, immunofluorescence assays in NRVMs showed that CAV1 abundance was higher in the HG group than in the CON group (Figure 1F-G).

### CAV1 deficiency attenuates DCM

**Figure 2:**
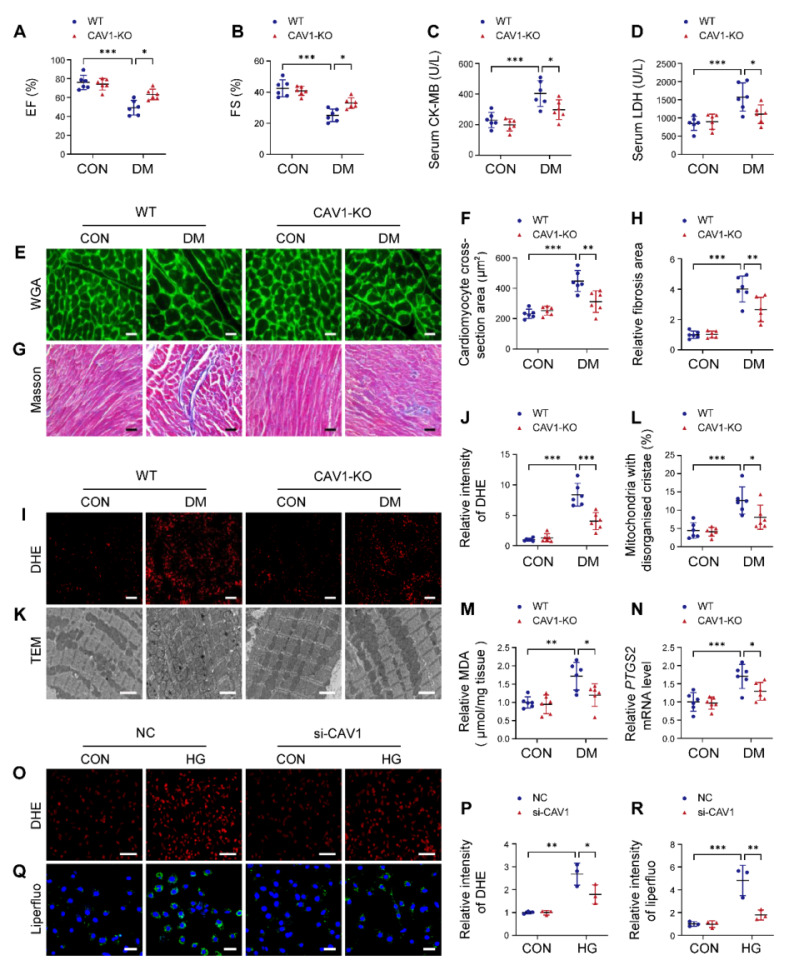
CAV1 knockout protects from diabetes-induced cardiac injury. (**A-B**) Echocardiographic assessment of ejection fraction (EF) and fractional shortening (FS). (**C-D**) Serum creatine kinase isoenzyme (CK-MB) and lactate dehydrogenase (LDH) levels were measured. (**E-F**) Representative wheat germ agglutinin (WGA) images and quantitative analysis of myocyte cross-sectional area. Scale bar = 20 μm. (**G-H**) Representative Masson's trichrome staining of myocardial fibers and quantification of the fibrosis area. Scale bar = 40 μm. (**I-J**) Dihydroethidium (DHE) staining and quantitative analysis of DHE fluorescence intensity. Scale bar = 40 μm. (**K-L**) Representative transmission electron microscopic images showing mitochondria from cardiac tissues, and quantitative analysis of the proportion of mitochondria with disorganised cristae. Scale bar = 2 µm. (**M**) Cardiac malondialdehyde (MDA) levels. (**N**) qRT-PCR analysis of ferroptosis marker *PTGS2* mRNA levels. (**O-P**) NRVMs were infected with si-NC or si-CAV1 and treated with or without HG for 12 hours. Intracellular reactive oxygen species (ROS) levels were detected using DHE staining. Scale bar = 100 µm. (**Q-R**) Representative fluorescence images of liperfluo staining. Scale bar = 40 μm. The data were presented as mean ± SD. ^*^P < 0.05, ^**^P < 0.01, ^***^ *P* < 0.001.

CAV1-KO mice were used to investigate the potential role of CAV1 in DCM, and the knockout efficiency in the heart tissue was determined (Figure S2A). Diabetic mice exhibited higher blood glucose levels and lower body weights than healthy controls, and CAV1 deficiency did not affect changes in blood glucose and body weight (Figure S2B-C). Echocardiographic analysis showed that ejection fraction (EF) and fractional shortening (FS) were increased in CAV1-KO diabetic mice compared with WT diabetic mice, indicating that the inhibition of CAV1 improved cardiac function (Figure 2A-B). We measured CK-MB and LDH levels in the serum and found that CK-MB and LDH were significantly decreased in CAV1-KO diabetic mice compared with WT diabetic mice (Figure 2C-D). Wheat germ agglutinin (WGA) staining showed that diabetes caused cardiomyocyte hypertrophy in WT mice, and the cross-sectional area of cardiomyocytes in CAV1-KO diabetic mice was smaller than that in WT diabetic mice (Figure 2E-F). Meanwhile, the heart weight/body weight ratio and the expression of hypertrophic gene *ANP* were decreased in CAV1-KO diabetic mice compared with WT diabetic mice (Figure S2D-E). Masson staining revealed that less cardiac fibrosis was observed in CAV1-KO diabetic mice than in WT diabetic mice (Figure 2G-H). CAV1 deficiency also reduced ROS production in diabetic hearts (Figure 2I-J). Transmission electron microscopy (TEM) revealed an increased proportion of mitochondria with disorganised and disrupted cristae in the hearts of WT diabetic mice, and that CAV1 deficiency mitigated abnormal mitochondrial structure in diabetic mice (Figure 2K-L). Furthermore, we found that cardiac MDA was significantly decreased in CAV1-KO diabetic mice compared with WT diabetic mice (Figure 2M). Similar results were observed for the mRNA expression of cardiac *PTGS2*, a putative molecular marker of ferroptosis (Figure 2N). To evaluate the effect of CAV1 on DCM *in vitro*, CAV1 was silenced in NRVMs using siRNA. The knockdown efficiency of CAV1 in NRVMs showed in Figure S3A. HG-induced excessive ROS production was reduced in NRVMs transfected with si-CAV1 compared with that in the NC (Figure 2O-P). Consistently, CAV1 knockdown markedly reduced HG-induced lipid peroxidation (Figure 2Q-R). Importantly, in contrast to untreated NRVMs, HG treatment led to an elevation in LDH levels, and silencing of CAV1 decreased LDH released from HG-treated NRVMs; this effect was reversed by the addition of erastin (an agonist of ferroptosis) (Figure S3B). Calcein-AM/PI staining indicated that PI-positive cells were increased in HG-treated NRVMs compared with control. Notably, silencing CAV1 significantly decreased HG-induced cell death, and this beneficial effect was abolished in NRVMs co-treated with erastin, indicating that silencing CAV1 alleviated HG-induced cardiomyocyte injury by inhibiting ferroptosis (Figure S3C-D). These data collectively indicated that CAV1 deficiency protected against diabetes-induced cardiac injury.

### Cardiac-specific overexpression of CAV1 aggravates DCM

**Figure 3:**
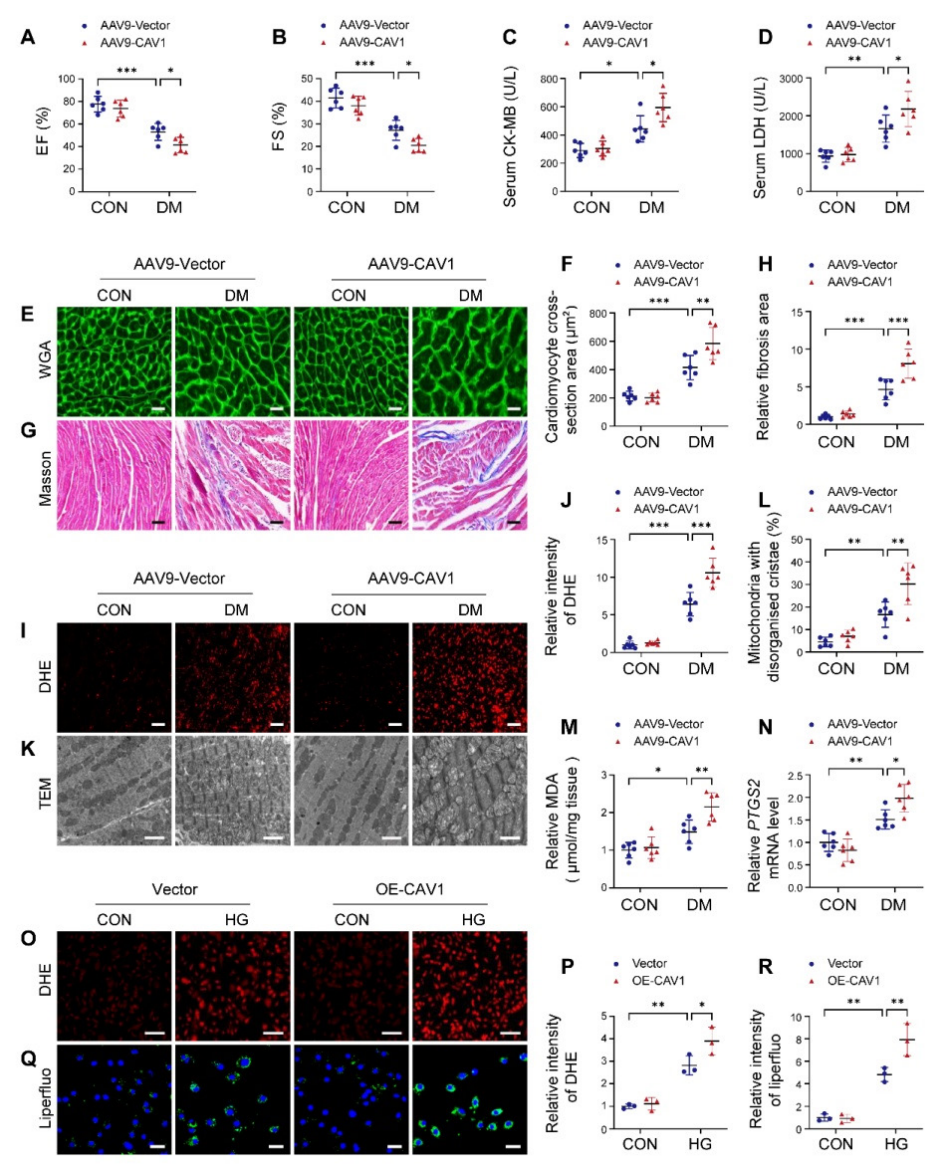
Cardiac-specific overexpression of CAV1 aggravates diabetes-induced cardiac injury. (**A-B**) Echocardiographic assessment of EF and FS. (**C-D**) Quantitative analysis of serum CK-MB and LDH. (**E-F**) Representative WGA images and quantitative analyses of myocyte cross-sectional area. Scale bar = 20 μm. (**G-H**) Fibrosis in cardiac tissues was determined using Masson's staining. Scale bar = 40 μm. (**I-J**) Representative DHE staining images and quantitative analysis. Scale bar = 40 μm. (**K-L**) Representative transmission electron microscopic images showing mitochondria from cardiac tissues, and quantitative analysis of the proportion of mitochondria with disorganised cristae. Scale bars = 2 µm. (**M**) Cardiac MDA levels. (**N**) qRT-PCR analysis of ferroptosis marker *PTGS2* mRNA levels. (**O-P**) NRVMs were infected with Lenti-Vector or Lenti-CAV1 and subsequently treated with or without HG for 12 hours. ROS level was detected using DHE staining. Scale bar = 100 µm. (**Q-R**) Representative fluorescence images of liperfluo staining. Scale bar = 40 µm. The data were presented as mean ± SD. ^*^P < 0.05, ^**^P < 0.01, ^***^ *P* < 0.001.

We used AAV9-mediated overexpression of CAV1 in mice. The overexpression efficiency of CAV1 in heart tissues was verified by western blotting (Figure S4A). Notably, cardiac-specific CAV1 overexpression had no significant effect on blood glucose and body weight in diabetic mice (Figure S4B-C). Cardiac-specific CAV1 overexpression further aggravated diabetes-induced cardiac dysfunction, as indicated by decreased EF and FS (Figure 3A-B). Moreover, cardiac-specific CAV1 overexpression increased serum CK-MB and LDH levels compared with AAV9-Vector diabetic mice (Figure 3C-D). In comparison with AAV9-Vector diabetic mice, AAV9-CAV1 diabetic mice exhibited an increase in the cardiomyocyte cross-sectional area and the collagen deposition (Figure 3E-H).

Additionally, AAV9-CAV1 diabetic mice produced more ROS and exacerbated the degree of mitochondrial damage than AAV9-Vector diabetic mice (Figure 3I-L). Meanwhile, MDA levels and *PTGS2* expression were increased in the hearts of AAV9-CAV1 diabetic mice compared with AAV9-Vector diabetic mice (Figure 3M-N). Consistent with the *in vivo* experimental results, CAV1 overexpression in NRVMs with lentivirus significantly increased the generation of cellular ROS and lipid peroxidation under HG condition compared with the Vector group (Figure 3O-R; S4D). Overexpression of CAV1 also led to a notable increase in PI-positive NRVMs when compared with HG alone, and this effect was inhibited by ferrostatin-1 treatment (an inhibitor of ferroptosis) (Figure S4E-F). These results demonstrated that cardiac-specific CAV1 overexpression exacerbated the progression of DCM.

### CAV1 directly interacts with NRF2

**Figure 4:**
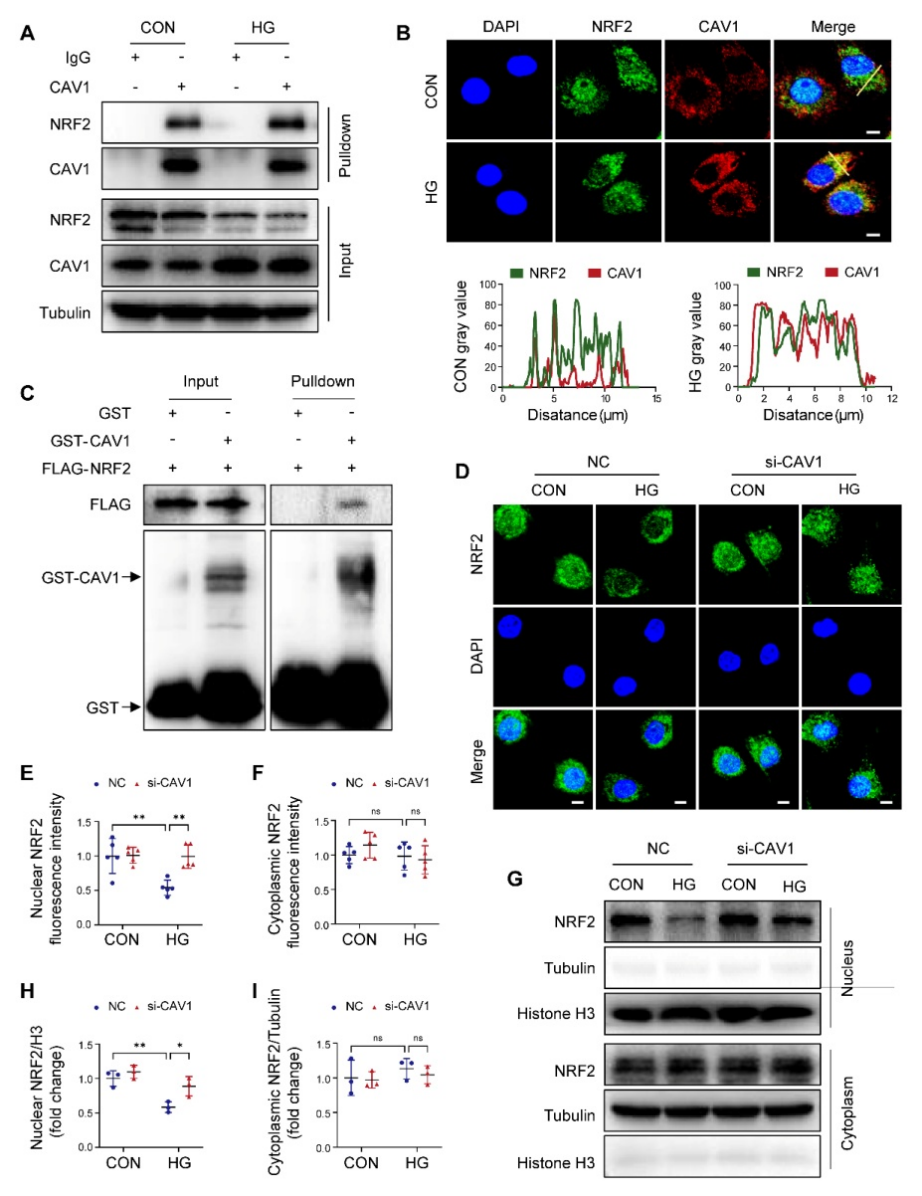
CAV1 interacts directly with nuclear factor erythroid 2- related factor 2 (NRF2). (**A**) NRVMs were treated with HG for 12 hours, following Co-IP with anti-CAV1. IgG was used as control for Co-IP. (**B**) Representative confocal microscopy images showing the colocalization of CAV1 (red), NRF2 (green) and DAPI (blue) in NRVMs. Scale bar = 5 μm. (**C**) Cellular extracts from HEK293T transfected with FLAG-NRF2 were pulled down with GST or GST-CAV1 conjugated to glutathione agarose beads and analyzed by western blotting with anti-FLAG or anti-GST antibody. (**D-F**) Representative immunofluorescence images of NRF2 (green) and DAPI (blue) in NRVMs infected with si-NC or si-CAV1 and subsequently treated with or without HG for 12 hours. Scale bar = 5 μm. (**G-I**) Representative western blot images and quantification of NRF2 in NRVMs. Data were expressed as mean ± SD. ^*^P < 0.05, ^**^P < 0.01, ns, indicates no significance.

NRF2 antioxidant system is crucial for regulating cellular redox homeostasis. Notably, previous studies reported that CAV1 inhibited the expression of antioxidant enzymes 35. By Co-IP analysis, we confirmed that CAV1 interacted with NRF2, and the interaction between CAV1 and NRF2 in HG-treated NRVMs was stronger than that in CON group (Figure 4A). Immunofluorescence showed that the co-localization of CAV1 and NRF2 in HG-treated NRVMs was higher than in the controls (Figure 4B). Furthermore, GST pull-down assay showed that FLAG-NRF2 could bind to GST-CAV1 but not to GST, suggesting that NRF2 directly interacted with CAV1 (Figure 4C). Moreover, immunofluorescence demonstrated that the abundance of nuclear NRF2 was weakened in HG-treated NRVMs, but silencing CAV1 significantly increased nuclear NRF2 without affecting cytoplasmic NRF2 under HG conditions (Figure 4D-F). Western blotting of nuclear and cytoplasmic extractions further confirmed that HG restricted the nuclear NRF2 protein levels in NRVMs compared with that of the control group, silencing CAV1 remarkably increased nuclear NRF2 expression, but had no significant impact on cytoplasmic NRF2 (Figure 4G-I). Conversely, compared to NRVMs treated with HG alone, overexpression of CAV1 further reduced the fluorescence intensity of NRF2 in the nucleus, although there was no significant difference (Figure S5A-C). Western blotting also confirmed that overexpression of CAV1 further decreased the nuclear NRF2 levels induced by HG, but had no significant effect on the cytoplasmic NRF2 levels (Figure S5D-F). Therefore, we speculated that HG promoted the expression of CAV1, causing an increased interaction between CAV1 and NRF2 and inhibition of NRF2 nuclear translocation. To verify the mechanisms* in vivo*, we analyzed the protein level of NRF2 in the cardiac tissues of mice. Western blotting indicated a significant reduction in NRF2 protein levels in the hearts of WT diabetic mice, which was recovered by CAV1 knockout (Figure S5G-H). NRF2 is an important transcription factor involved in the regulation of antioxidant gene expression. Next, we assayed NRF2-induced antioxidant genes, specifically *GPX4* and *HO-1*. We observed an upregulation of *GPX4* and *HO-1* in the hearts of CAV1- KO diabetic mice when compared to WT diabetic mice, suggesting that the absence of CAV1 facilitated the nuclear translocation of NRF2 and activation of antioxidant genes *in vivo* (Figure S5I-J). Collectively, our data demonstrated that CAV1 interacted with NRF2 and inhibited NRF2 nuclear translocation.

### NRF2 plays a beneficial role in DCM

**Figure 5:**
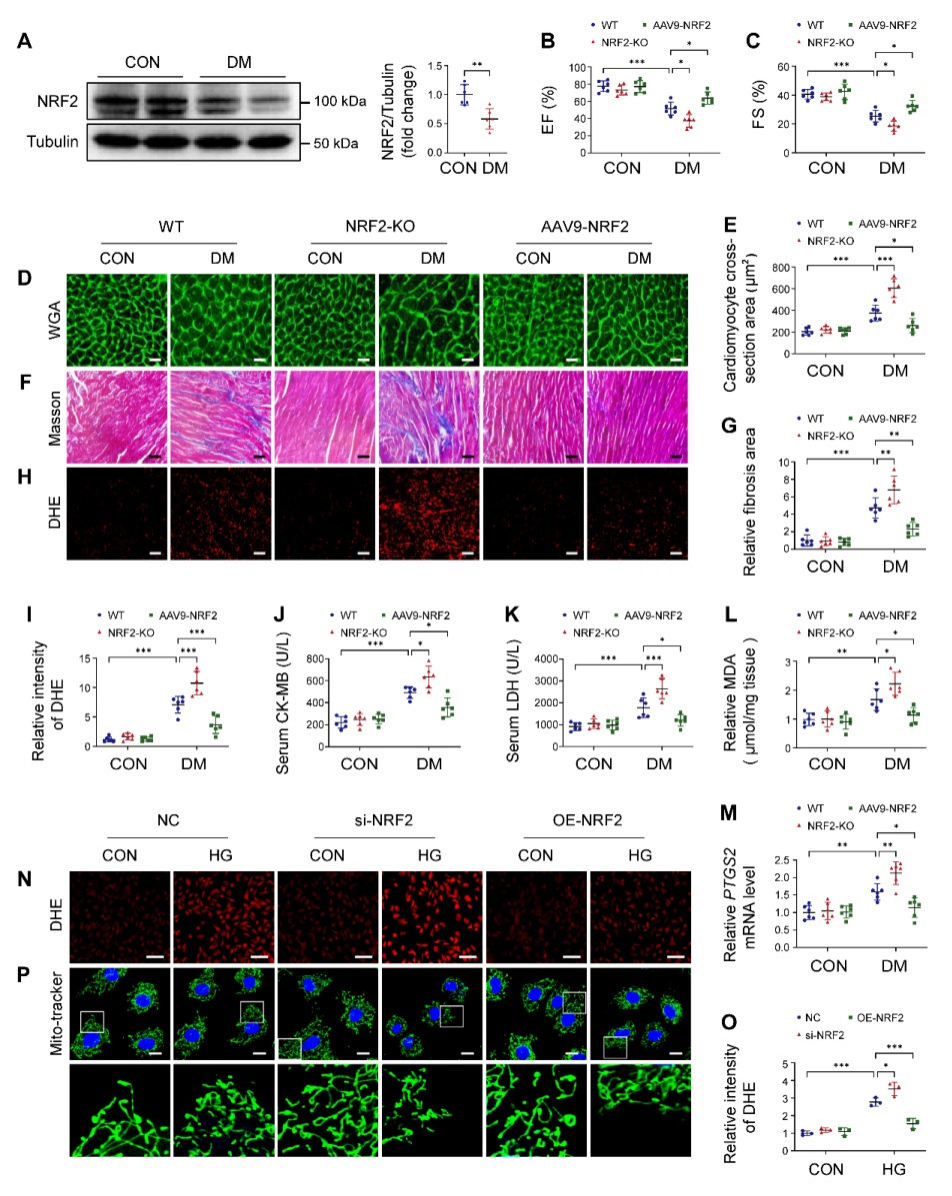
NRF2 plays a crucial role in DCM. (**A**) Western blotting of NRF2 expression in heart tissue. (**B-C**) Echocardiographic assessment of EF and FS. (**D-E**) Representative WGA images and quantitative analyses of myocyte cross-sectional area. Scale bar = 20 μm. (**F-G**) Representative Masson's trichrome staining of myocardial fibers and quantification of the fibrosis area. Scale bar = 40 μm. (**H-I**) Representative DHE staining images and quantitative analysis of DHE fluorescence intensity. Scale bar = 40 μm. (**J-K**) Quantitative analysis of serum CK-MB and LDH levels. (**L**) Cardiac MDA levels. (**M**) Relative mRNA levels of *PTGS2* in mouse heart samples. (**N-O**) NRVMs were infected with si-NRF2 or Lenti-NRF2, and subsequently treated with or without HG for 12 hours. Intracellular ROS level was detected using DHE staining. Scale bar = 100 μm. (**P**) Mitochondrial network was detected using Mito-tracker staining. Scale bar = 20 μm. Data were expressed as mean ± SD. ^*^P < 0.05, ^**^P < 0.01, ^***^ *P* < 0.001.

The NRF2-KO and cardiac-specific NRF2 overexpression mice were used to study the role of NRF2 in DCM. As shown in Figure S6A, the knockout efficiency of NRF2 was verified. AAV9-mediated NRF2 overexpression in heart was confirmed, but no significant differences were observed in other organs (Figure S6B-D). NRF2 deletion or overexpression had no significant effects on blood glucose and body weight (Figure S6E-F). Western blotting of cardiac tissues revealed a significant decrease of NRF2 levels in diabetic mice (Figure 5A). Echocardiography results showed that NRF2-KO diabetic mice developed more severe cardiac dysfunction, manifesting as decreased EF and FS than WT diabetic mice (Figure 5B-C). Pathological examination confirmed that the absence of NRF2 led to more severe hypertrophy, interstitial fibrosis and excessive ROS production in diabetic mice (Figure 5D-I). Meanwhile, we found that serum CK-MB and LDH, cardiac MDA, and mRNA levels of *PTGS2* in NRF2-KO diabetic mice were higher than those in WT diabetic mice (Figure 5J-M). Conversely, compared to WT diabetic mice, cardiac-specific overexpression NRF2 protected diabetic mice from cardiac structural and functional abnormalities, as reflected by increased EF and FS, attenuated hypertrophy and interstitial fibrosis, and reduced levels of ROS (Figure 5B-I). AAV9-NRF2 diabetic mice significantly reduced serum CK-MB and LDH, cardiac MDA, and *PTGS2* expression compared with WT diabetic mice (Figure 5J-M). In *in vitro* experiments, NRF2 silencing in NRVMs significantly caused an increase in ROS levels and exacerbated mitochondrial fragmentation under HG conditions compared with the NC group (Figure 5N-P; S7A). In contrast, NRF2 overexpression reversed HG-induced ROS production and mitochondrial alterations (Figure 5N-P; S7B). Collectively, these data suggested that NRF2 was pivotal for maintaining cardiac function in DCM.

### Beneficial effect of CAV1 knockout is dependent on NRF2

**Figure 6:**
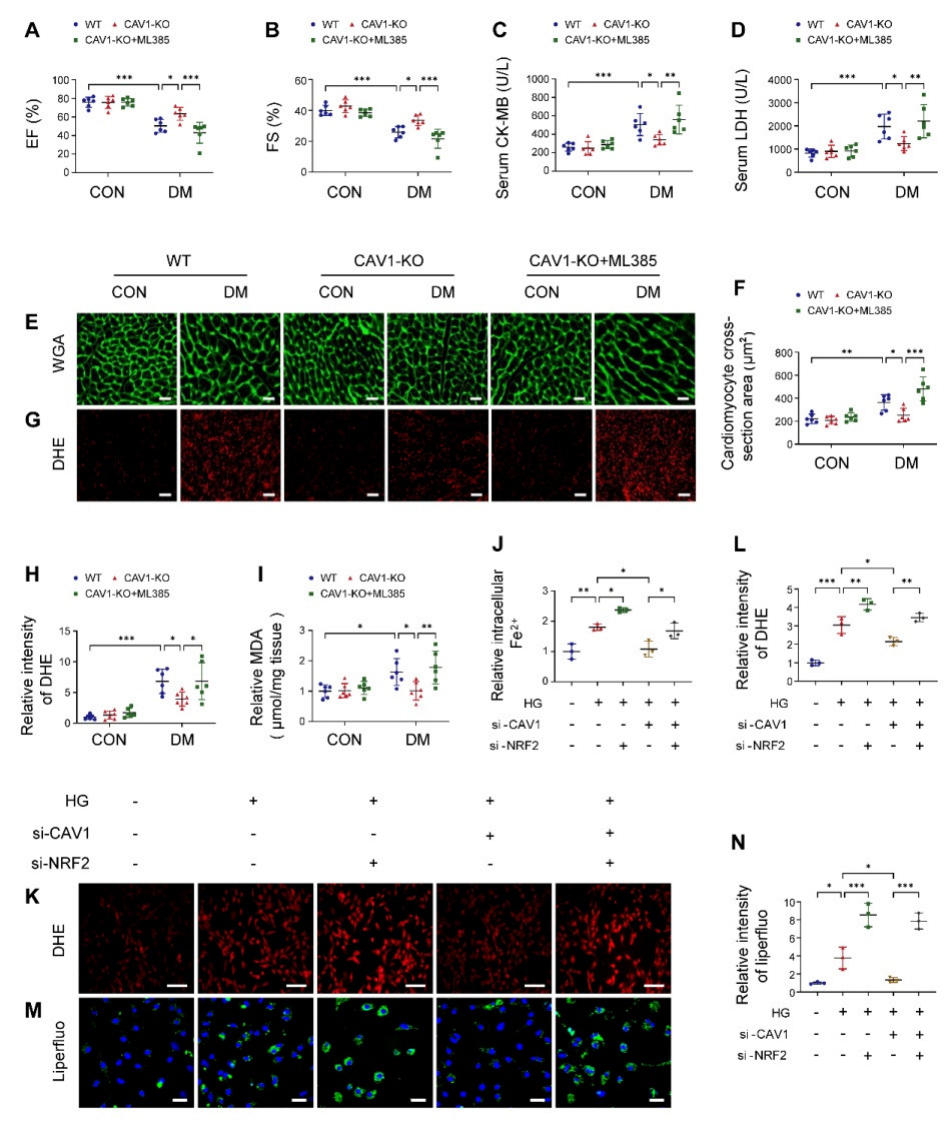
Beneficial effect of CAV1 knockout is dependent on NRF2. (**A-B**) Echocardiographic assessment of EF and FS. (**C-D**) Serum CK-MB and LDH levels. (**E-F**) Representative WGA images and quantitative analyses of myocyte cross-sectional area. Scale bar = 20 μm. (**G-H**) Representative DHE staining images and quantitative analysis of DHE fluorescence intensity. Scale bar = 40 μm. (**I**) Cardiac MDA levels. (**J**) NRVMs were transfected with si-CAV1 and/or si-NRF2, and exposed to HG for 12 hours. The intracellular ferrous ions were examined. (**K-L**) Intracellular ROS level was detected by DHE staining. Scale bar = 100 μm. (**M-N**) Lipid peroxides were measured by liperfluo staining. Scale bar = 40 μm. Data were expressed as mean ± SD. ^*^P < 0.05, ^**^P < 0.01, ^***^ *P* < 0.001.

To determine the necessity of NRF2 in CAV1-mediated cardiac injury, we administered a specific NRF2 inhibitor (ML385) to CAV1-KO mice. ML385 effectively inhibited the expression of NRF2 *in vivo* (Figure S8A). ML385 did not alter the blood glucose and body weight of diabetic mice (Figure S8B-C). Cardiac functional tests revealed that ML385 reversed the protective effects of CAV1 deficiency in diabetes-induced cardiac dysfunction, as reflected by decreased EF and FS (Figure 6A-B). In addition, ML385-treated CAV1-KO diabetic mice significantly increased serum CK-MB and LDH levels, promoted hypertrophy and oxidative stress compared with untreated CAV1-KO diabetic mice (Figure 6C-H). Similarly, cardiac MDA were increased significantly when CAV1-KO diabetic mice treated with ML385 (Figure 6I). Consistent with our *in vivo* results, we observed that silencing NRF2 enhanced HG-induced increases in ferrous ions, intracellular ROS, and lipid peroxidation in CAV1-knockdown NRVMs (Figure 6J-N). Collectively, these results supported that CAV1 regulated DCM in an NRF2-dependent manner.

### NRF2 binds to the GCLC promoter and activates its transcription

**Figure 7:**
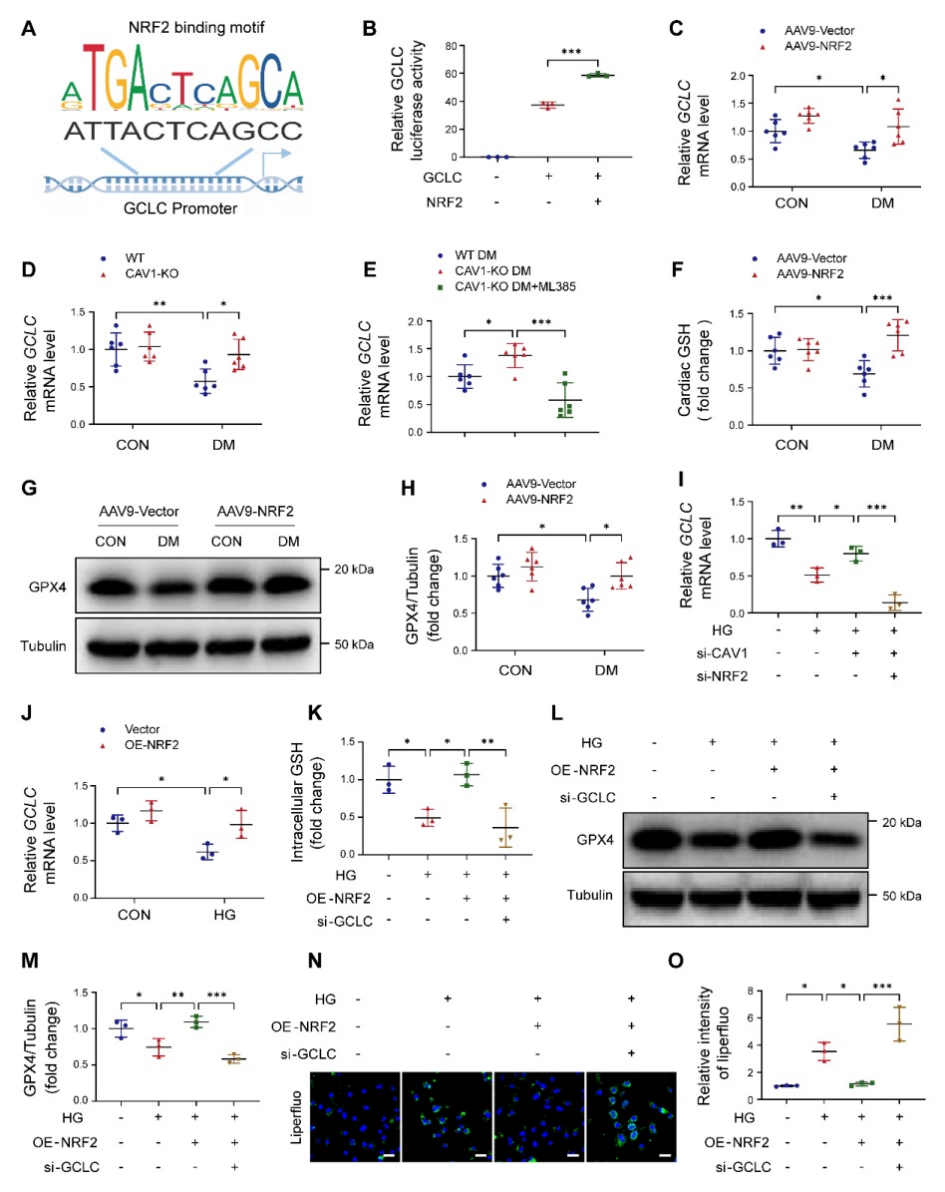
NRF2 binds to the glutamate cysteine ligase catalytic subunit (GCLC) promoter and activates its transcription. (**A**) DNA-binding motifs of NRF2 according to the JASPAR database. (**B**) Dual luciferase reported assay experiment. (**C-E**) mRNA levels of cardiac *GCLC* were determined using qRT-PCR. (**F**) Assessment of glutathione (GSH) from heart tissue. (**G-H**) Representative western blotting of glutathione peroxidase-4 (GPX4) expression in cardiac tissues. (**I-J**) qRT-PCR analysis of *GCLC* mRNA levels. (**K**) NRVMs were infected with Lenti-NRF2 alone or Lenti-NRF2 combined with si-GCLC, then exposed to HG for 12 hours. Assessment of GSH from NRVMs. (**L-M**) Representative western blotting of GPX4 in NRVMs. (**N-O**) Lipid peroxides were measured by liperfluo staining. Scale bar = 40 μm. Data were expressed as mean ± SD. ^*^P < 0.05, ^**^P < 0.01, ^***^ *P* < 0.001.

GCLC is the catalytic subunit of GCL, which is the rate-limiting enzyme in GSH synthesis. We used the JASPAR database to predict NRF2-binding sites in the proximal region of the GCLC promoter, and discovered two identical NRF2-binding sites (ATTACTCAGCC) located at -460 to -470 and -646 to -656 in the GCLC promoter region (Figure 7A). Dual-luciferase reporter assays further demonstrated that NRF2 bound to the promoter region of GCLC and regulated its expression (Figure 7B). NRF2 overexpression significantly increased *GCLC* mRNA expression in the cardiac tissues of diabetic mice (Figure 7C). CAV1 knockout also enhanced the *GCLC* mRNA levels in the hearts of diabetic mice (Figure 7D). In contrast, ML385-treated CAV1-KO diabetic mice significantly decreased the *GCLC* mRNA levels when compared to untreated CAV1-KO diabetic mice (Figure 7E). Furthermore, AAV9-NRF2 diabetic mice significantly promoted cardiac GSH production and GPX4 levels compared with AAV9-vector diabetic mice (Figure 7F-H). For *in vitro* experiments, CAV1 silencing significantly up-regulated *GCLC* expression in HG-treated NRVMs compared with HG treatment alone, but this effect was reversed by silencing NRF2 (Figure 7I). NRF2 overexpression markedly increased the expression of *GCLC* after HG stimulation (Figure 7J). Further, NRF2 overexpression increased the intracellular GSH and GPX4 and reduced lipid peroxidation in HG-treated NRVMs; however, both NRF2 overexpression and GCLC silencing exhibited a reduced amount of GSH and GPX4, and an increase in lipid peroxidation production when compared with the overexpression of NRF2 alone (Figure 7K-O; S8D-E). These results suggested that CAV1/NRF2 signaling regulated cardiomyocyte ferroptosis by regulating the transcription of GCLC.

### Targeting CAV1 protects against DCM

**Figure 8:**
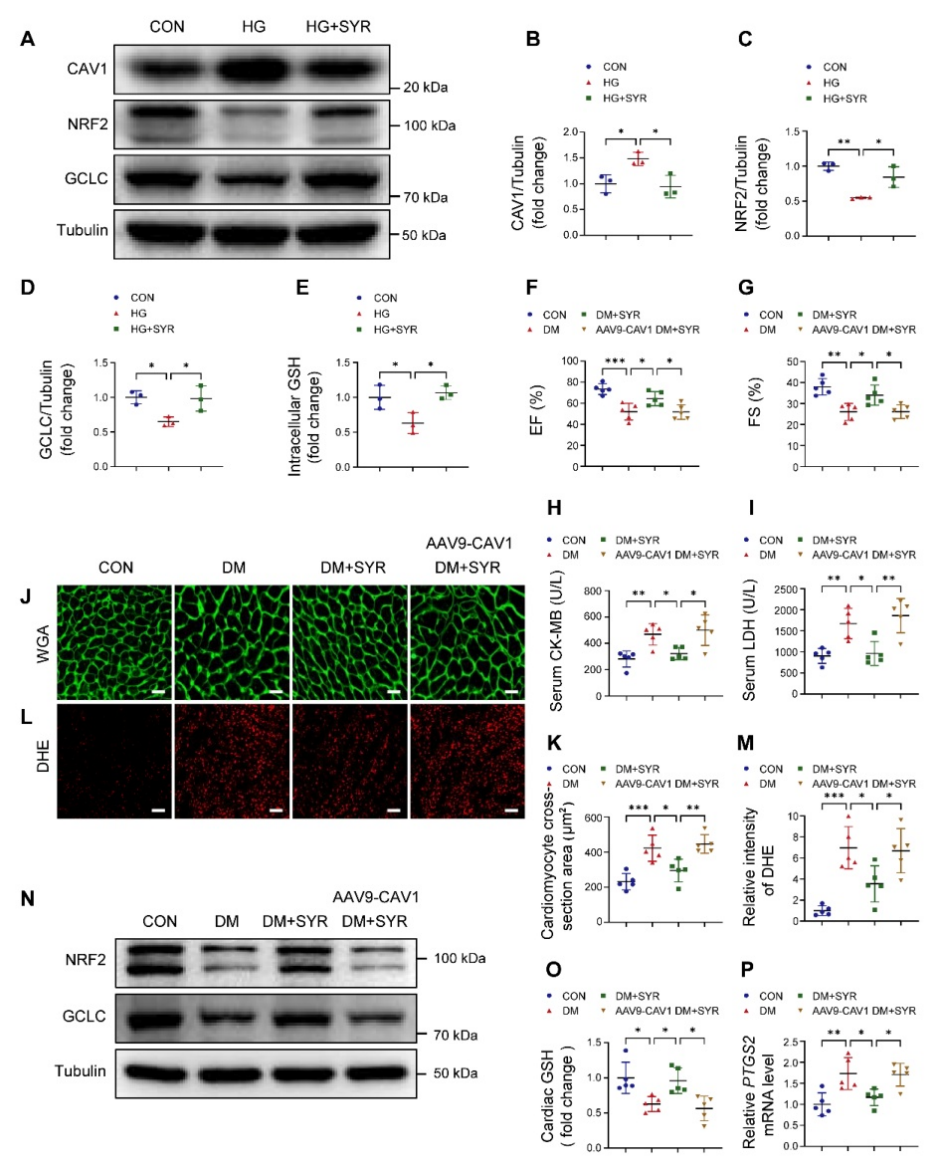
Syringaresinol (SYR) protects against DCM by inhibiting CAV1. (**A-D**) NRVMs were pretreated with SYR (50 μM) for 2 hours and exposed to HG for 12 hours. Western blotting of CAV1, NRF2, and GCLC. (**E**) Measurement of GSH in NRVMs. (**F-G**) Echocardiographic assessment of EF and FS. (**H-I**) Serum CK-MB and LDH levels. (**J-K**) Representative WGA images and quantitative analyses of myocyte cross-sectional area. Scale bar = 20 μm. (**L-M**) Representative DHE staining images and quantitative analysis of DHE fluorescence intensity. Scale bar = 40 μm. (**N**) Western blotting of NRF2 and GCLC in cardiac tissues. (**O**) Measurement of cardiac GSH levels. (**P**) qRT-PCR analysis of ferroptosis marker *PTGS2* mRNA expression. Data were expressed as mean ± SD. ^*^P < 0.05, ^**^P < 0.01, ^***^ *P* < 0.001.

Previous studies have reported that SYR alleviates diabetes-mediated cardiac injury 30. Molecular docking analysis indicated that SYR interacted with CAV1 (Figure S9A). Western blotting confirmed that SYR did decrease HG-induced CAV1 expression and positively upregulated the abundance of NRF2 and GCLC when compared to HG alone-treated NRVMs (Figure 8A-D). Moreover, SYR treatment elevated intracellular GSH levels under HG conditions (Figure 8E). In *in vivo* experiments. SYR administration did not affect the blood glucose and body weight in diabetic mice (Figure S9B-C). SYR treatment improved EF and FS, reduced the release of CK-MB and LDH, and prevented cardiac hypertrophy and oxidative stress in diabetic mice. However, these protective effects were abolished when simultaneously cardiac-specific overexpression of CAV1 in diabetic mice (Figure 8F-M). In addition, SYR administration significantly upregulated the expression of NRF2 and GCLC, while cardiac-specific overexpression of CAV1 suppressed these effects (Figure 8N; S9D-E). The increased cardiac GSH in SYR-fed diabetic mice was prevented by overexpressing CAV1 (Figure 8O). Meanwhile, SYR treatment significantly reduced the expression of *PTGS2* in the cardiac tissue of diabetic mice, which was reversed by the overexpression of CAV1 (Figure 8P). These results suggested that CAV1 may be a promising therapeutic target for DCM.

## Discussion

Diabetes is a global health challenge owing to its serious complications, among which DCM is one of the most common and disabling complications 36, 37. The precise mechanisms involved in the development of DCM require further explorations to facilitate the discovery of clinically effective targets for preventing its progression. Several studies have reported that ferroptosis is essential for the pathogenesis of DCM 19. Increased lipid peroxidation during ferroptosis promotes oxidative stress and damages myocardial cells, leading to cardiac dysfunction 18, 38. In this study, we identified CAV1 as a potential therapeutic target for DCM. CAV1 deficiency significantly attenuated diabetes-induced myocardial hypertrophy, fibrosis, oxidative stress, and cardiomyocyte ferroptosis and improved cardiac dysfunction by activating the NRF2/GCLC signaling pathway.

CAV1 is a structural protein component of caveolae and involved in various cellular processes, including signal transduction 39, 40. CAV1 is composed of four domains, the N-terminal domain includes the tyrosine (Tyr14) phosphorylation site, which can respond to a variety of stimuli; the caveolin-scaffolding domain mediates direct interactions between CAV1 and multiple signaling molecules; the transmembrane domain interacts with membrane lipids; the C-terminal domain contains the palmitoylation site, which contributes to plasma membrane localization 41, 42. In atherosclerosis, CAV1 interacts with the ATG5-ATG12 complex and influences the cellular localization of autophagosome components; CAV1 deficiency protects against the progression of atherosclerosis by increasing autophagy 6. Depletion of CAV1 attenuates hepatic fibrosis by promoting SQSTM1-mediated PFKL degradation in hepatic stellate cells 43. CAV1 knockdown enhances the therapeutic sensitivity of lung cancer to cisplatin-induced apoptosis by inhibiting parkin-related mitophagy and activating ROCK1 pathway 44. Notably, we found that CAV1 was upregulated in the cardiac tissues of diabetic mice (Figure 1). *In vitro*, we established DCM by culturing NRVMs with 33mM glucose. We found that CAV1 expression showed an increasing trend in HG-cultured NRVMs after 6 hours, with the most significant increase at 12 or 18 hours. However, compared to the HG-treated 12 hours group, there was a decrease trend in CAV1 expression after 24 hours, yet it remained above the baseline levels (Figure 1). Therefore, we selected the 12-hour HG treatment for our subsequent experiments. CAV1 deficiency significantly attenuated diabetes-induced cardiac systolic dysfunction and myocardial injury (Figure 2), and that cardiac-specific overexpression of CAV1 accelerated the progression of DCM (Figure 3). Previous studies had revealed that diabetic mice exhibit both systolic and diastolic dysfunction 19, 45. However, our study did not include the relevant indices for evaluating diastolic function, which would have provided a more comprehensive understanding of cardiac function. Our future research will address this limitation to elucidate cardiac diastolic function in diabetic wild-type and CAV1-knockout/overexpressing mice. Notably, Li et al. first demonstrated that CAV1 could directly interact with NRF2 and inhibit its transcriptional activity, thereby inhibiting the expression of antioxidant enzymes HO-1 and GCLC in lung epithelial Beas-2B cells 46. Peter C Hart et al. found that CAV1 reduced MnSOD expression via suppressing NRF2 activity in breast cancer cell (MCF7) 47. Xu et al. discovered that the association between CAV1 and NRF2 was intensified in human hepatocyte L02 cells exposed to aflatoxin B1, and that CAV1 knockdown promoted NRF2 nucleus translocation and attenuated AFB1-induced MDA and ROS levels 48. However, the interaction between CAV1 and NRF2 in DCM remains unstudied. Here, we confirmed the direct interaction between CAV1 and NRF2 using Co-IP and GST pull-down assays and found that this interaction was more significant in HG-treated NRVMs. Fluorescence colocalization of CAV1 and NRF2 in NRVMs confirmed that CAV1 mainly colocalized with NRF2 in the cytoplasm, causing reduced NRF2 nuclear translocation (Figure 4). Utilizing CAV1 mutations or peptides to disrupt the interaction between CAV1 and NRF2 can provide a more comprehensive conclusion in DCM. Our future studies will be aimed at solving this problem. Our data showed that NRF2 deficiency aggravated diabetes-induced myocardial hypertrophy, fibrosis, oxidative stress, and cardiomyocyte ferroptosis; however, cardiac-specific NRF2 overexpression by AAV9 greatly slowed the progression of DCM (Figure 5). These results confirmed that NRF2 was crucial in the protection against DCM. Furthermore, we administered the NRF2 inhibitor ML385 to CAV1-KO mice and confirmed that the protective effect of CAV1 knockout on DCM was dependent on NRF2 (Figure 6). KEAP1 is a negative regulator of NRF2, inhibiting its nuclear translocation and promoting its ubiquitination degradation through direct binding to NRF2. Limited researches indicated that silencing CAV1 decreased the protein levels of KEAP1 and enhanced the NRF2-ARE transcriptional binding in polychlorinated biphenyls-treated endothelial cells 49. Furthermore, knockdown of CAV1 disrupted the interaction between NRF2 and KEAP1, resulting in an elevation in NRF2 transcriptional activity in lung epithelial Beas-2B cells 46. The impact of CAV1 on KEAP1 in DCM deserves further investigation.

GCLC is the catalytic subunit of GCL, a vital rate-limiting enzyme in GSH biosynthesis 50. Luo et al. reported that silencing GCLC accelerated ferroptosis and decreased the proliferation and invasion of lung adenocarcinoma cells 51. Yang et al. found that ACTL6A upregulated GCLC as a co-transcription factor with NRF2, reducing ROS production and inhibiting ferroptosis in gastric cancer 22. Han et al. proved that upregulation of GCLC promoted GSH synthesis and prevented CD36-mediated ferroptosis, thus enhancing T cell anti-tumor function 52. For the first time, our results revealed that NRF2 bound to the GCLC promoter, and that knockdown of CAV1 or overexpression of NRF2 activated the transcription of GCLC, promoting the biosynthesis of GSH and inhibiting ferroptosis in DCM (Figure 7). SYR is a natural plant-derived polyphenolic compound that possesses excellent antioxidant activity 28, 53. Our previous study reported that SYR protected against DCM by activating NRF2-mediated antioxidant pathway 30. Here, we found that treatment with SYR significantly increased EF and FS, reduced diabetes-induced cardiac hypertrophy, oxidative stress, and cardiomyocyte ferroptosis. However, these beneficial effects were nearly absent when SYR administrated cardiac-specific overexpression CAV1 diabetic mice. Meanwhile, SYR significantly reduced CAV1 expression and upregulated the downstream target NRF2/GCLC signaling in HG-treated NRVMs and the cardiac tissue of diabetic mice (Figure 8).

To our knowledge, this is the first demonstration of the relationship between CAV1 and NRF2 in DCM. We showed that inhibition of CAV1 alleviated diabetes-induced myocardial injury by activating NRF2/GCLC signaling. Therefore, CAV1 may be a promising therapeutic target for DCM.

## Supplementary Material

Supplementary figures and tables.
