# Supplementary material for: Inhibition of CAV1 attenuates diabetic cardiomyopathy through reducing ferroptosis via activating NRF2/GCLC signaling pathway
Source: Theranostics. 2025 Mar 31;15(11):4989–5006. doi: 10.7150/thno.107367 (PMC12036865; doi:10.7150/thno.107367)
Supplement: Supplementary file 1 — Supplementary figures and tables. [file thnov15p4989s1.pdf]

Table S1. Primers used for CAV1 genotyping

Primer	Sequence (5'→3')	Primer type
P1	TTTGCCACCCCACAGTCATT	Forward
P2	AGGCAGTTGAGGTTGTTGGT	Reverse
P3	TGCCACCCCACAGTCATTTC	Forward
P4	GATGAGTGCCATTGGGATGC	Reverse

Table S2. Primers used for NRF2 genotyping

Primer	Sequence (5'→3')	Primer type
P1	CATCCCGTCCTTTGGCTGAG	Forward
P2	GAGGGGGTTGGAAAGAGATGTATG	Reverse
P3	GGTTAGCAGCGCAGGAGCATTAGT	Forward
P4	TACAGGCAAGAAGAAGGCATCAGA	Reverse

Table S3. Sequences of the primers

Gene	Species	Forward	Reverse
CAV1	rat	CATTCCCGACTCTTACGC	TTTTGACACCCCTCCCT
GCLC	rat	ACATCTACCACGCAGTCAAGGAC	GAACATCGCCGCCATTCAGTAAC
PTGS2	mouse	CTGGTGCCTGGTCTGATGATGTATG	GGATGCTCCTGCTTGAGTATGTCG
GCLC	mouse	ACTTCCTCATTCCGCTGTCCAAG	GCCGCCTTTGCAGATGTCTTTC
ANP	mouse	GCTTCCAGGCCATATTGGAG	GGGGGCATGACCTCATCTT
GPX4	mouse	ATAAGAACGGCTGCGTGGTGAAG	TAGAGATAGCACGGCAGGTCCTTC
HO-1	mouse	TCCTTGTACCATATCTACACGG	GAGACGCTTTACATAGTGCTGT

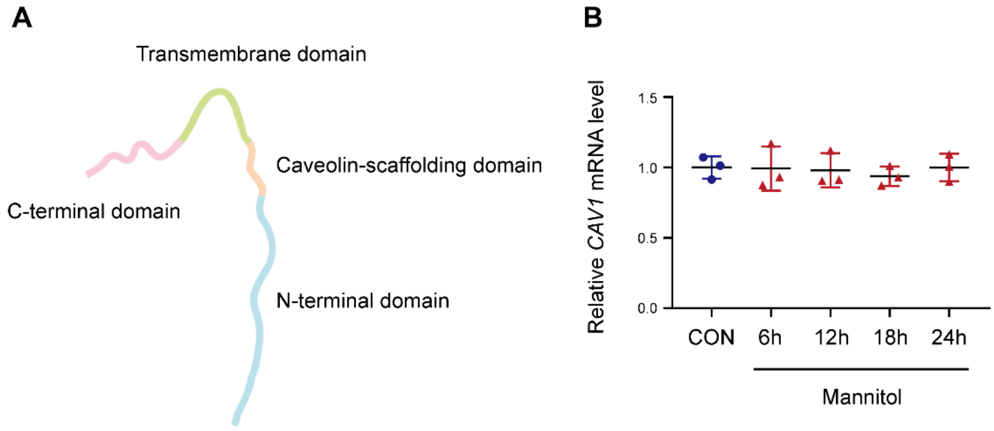

Figure S1. (**A**) Schematic diagram of CAV1 domains. (**B**) NRVMs were treated with mannitol for 6, 12, 18 and 24 hours. qRT-PCR analysis of *CAV1* mRNA expression.

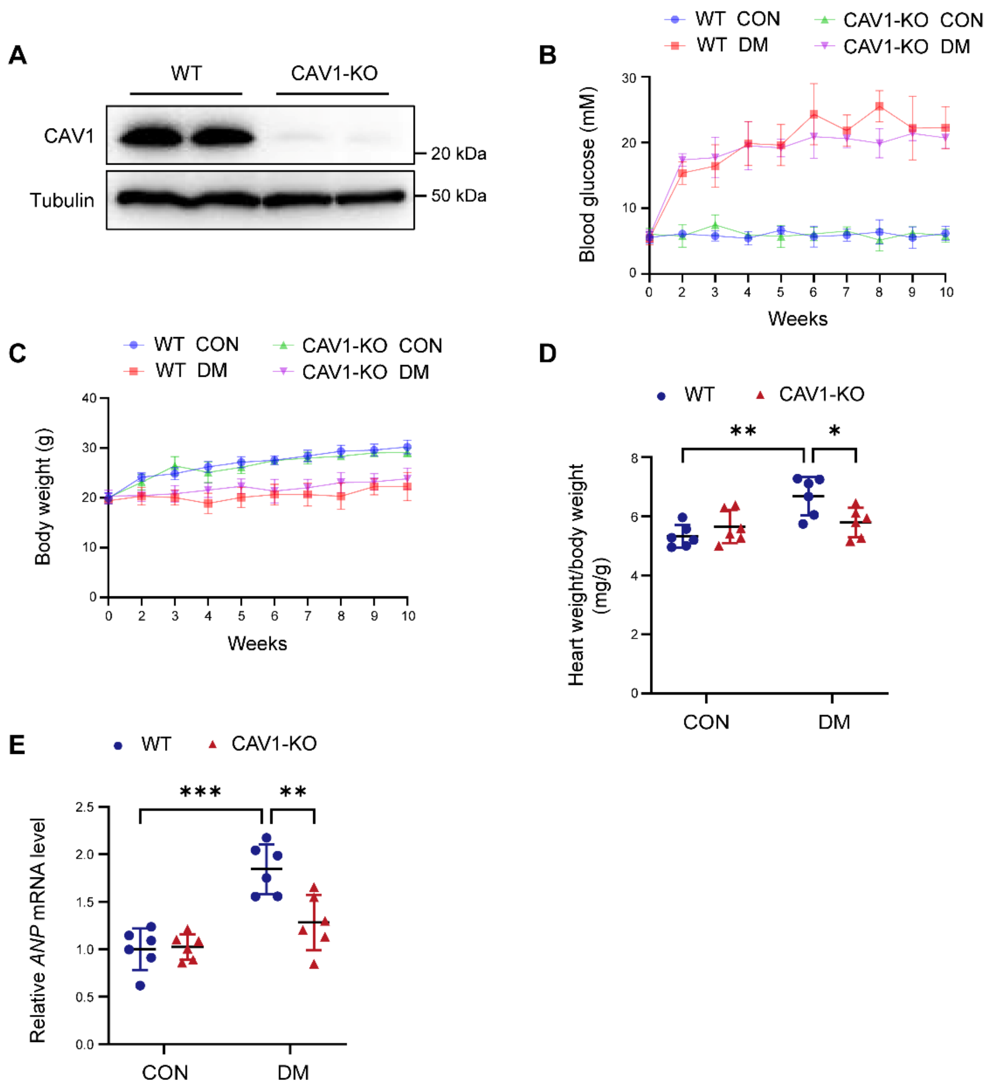

Figure S2. (**A**) Western blotting of CAV1 expression in heart samples from WT or CAV1-KO mice. (**B**) The blood glucose was measured weekly. (**C**) The body weight was measured weekly. (**D**) Ratio of heart weight (mg) to body weight (g). (**E**) mRNA level of *ANP* in heart tissues of mice. The data were presented as mean ± SD, $^{*}P < 0.05$. $^{**}P < 0.01$, $^{***}P < 0.001$.

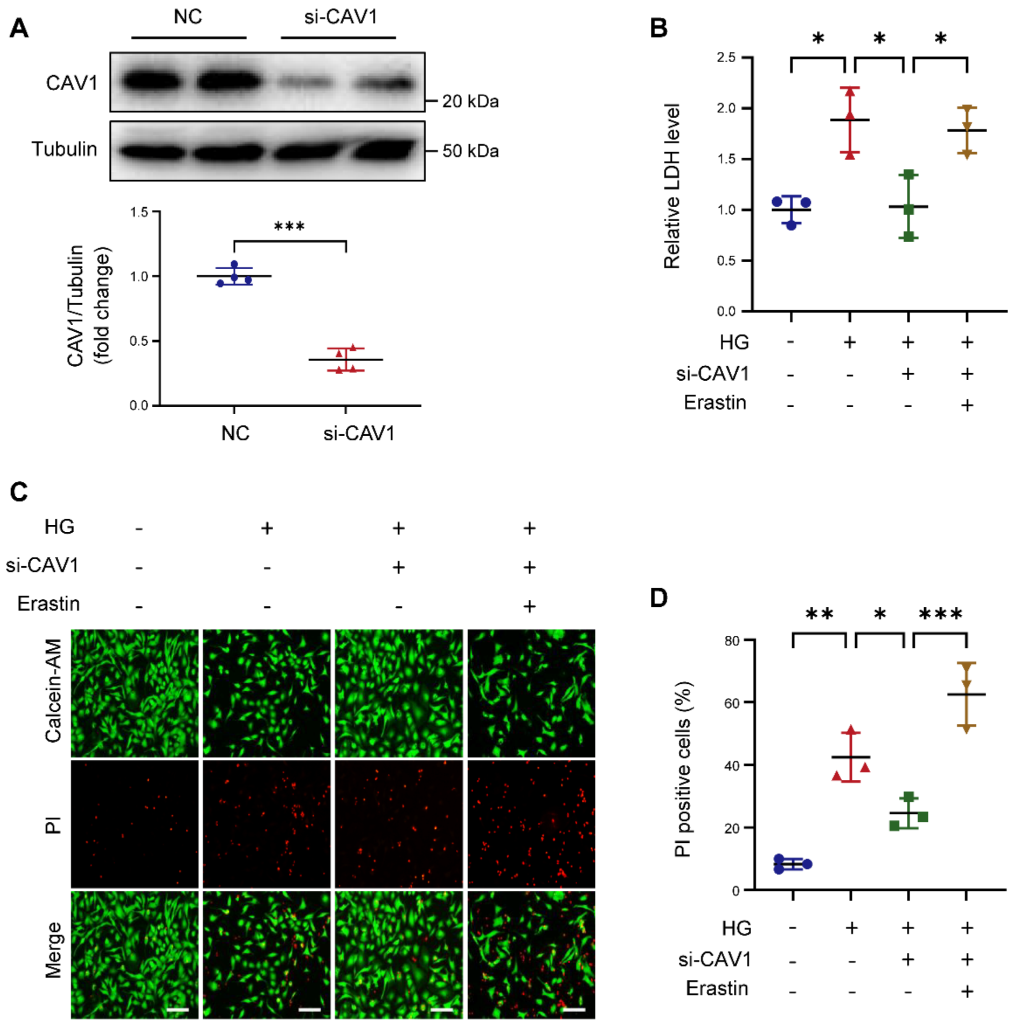

Figure S3. (**A**) Representative western blotting images and quantification of CAV1 in NRVMs transfected with si-NC or si-CAV1 for 48 hours. (**B**) NRVMs were infected with si-CAV1 alone or si-CAV1 combined with erastin, and subsequently treated with HG for 24 hours. LDH were measured. (**C-D**) Representative images of Calcein-AM/PI staining. Scale bar = 100 μm. The data were presented as mean ± SD, $^{*}P < 0.05$. $^{**}P < 0.01$, $^{***}P < 0.001$.

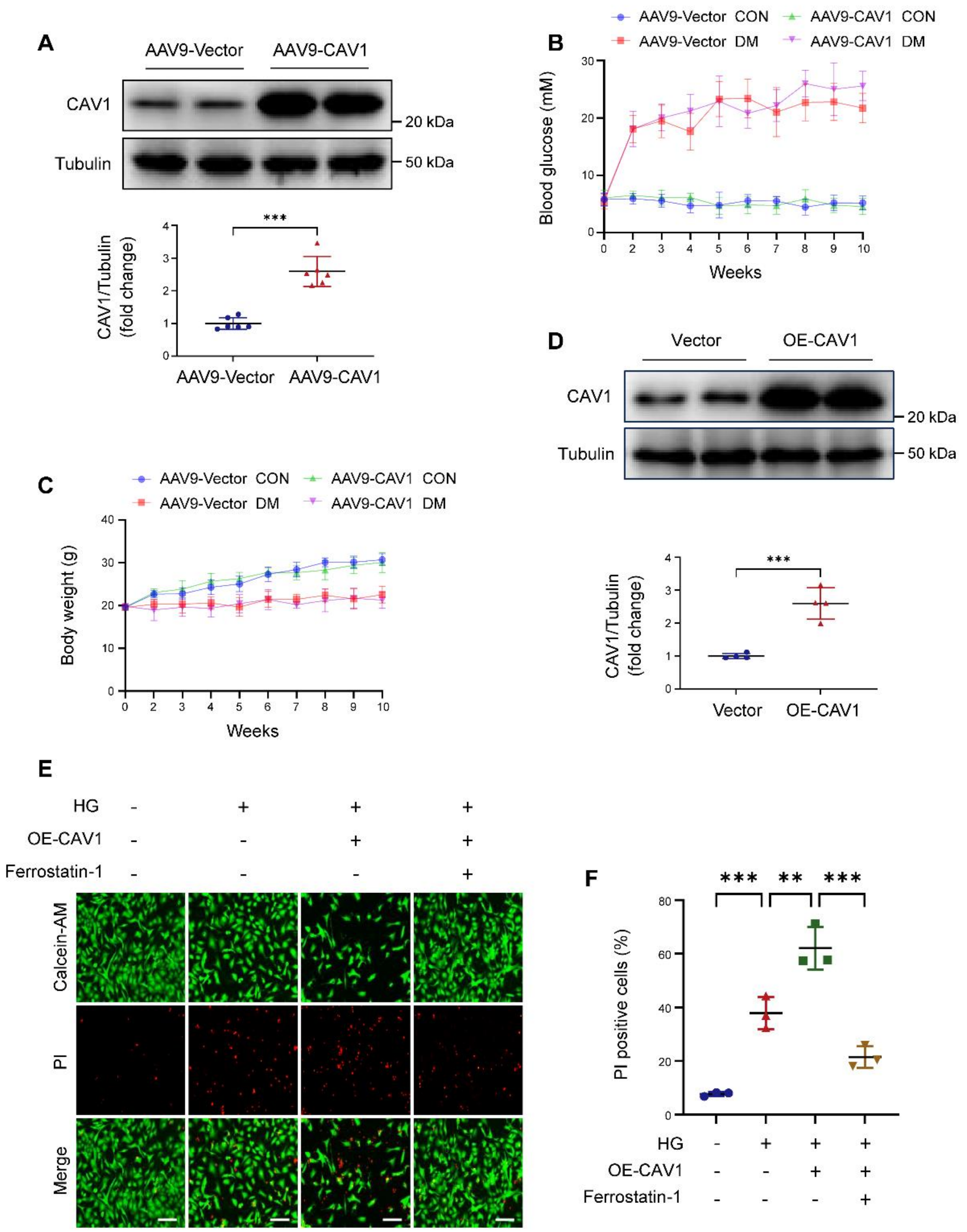

Figure S4. (**A**) Representative western blotting images and quantitative analysis of CAV1 in mouse cardiac tissues. (**B**) The blood glucose was measured weekly. (**C**) The body weight was measured weekly. (**D**) Representative western blotting and quantification of CAV1 in NRVMs infected with Lenti-Vector or Lenti-CAV1 for 48 hours. (**E-F**) Calcein-AM/PI staining in NRVMs infected with Lenti-CAV1 alone or Lenti-CAV1 combined with ferrostatin-1, and subsequently treated with HG for 24 hours. Scale bar = 100 μm. Data were presented as mean ± SD. $^{**}P < 0.01$, $^{***}P < 0.001$.

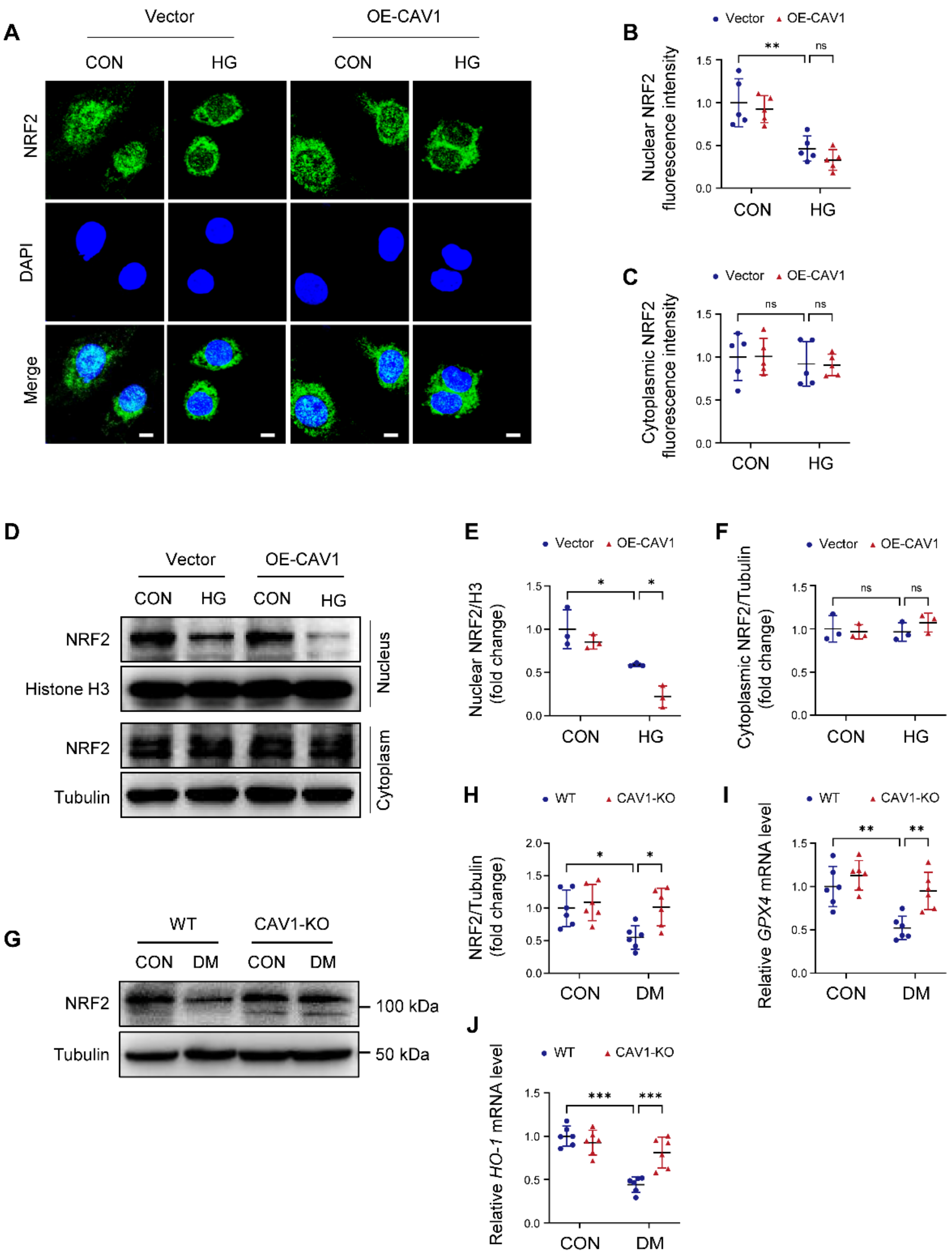

Figure S5. (**A-C**) Representative immunofluorescence images of NRF2 (green) and DAPI (blue) in NRVMs infected with Lenti-Vector or Lenti-CAV1 and subsequently treated with or without HG for 12 hours. Scale bar = 5 µm. (**D-F**) Western blotting and quantification of NRF2 in NRVMs. (**G-H**) Representative immunoblotting images and quantification of NRF2 in heart tissues. (**I-J**) mRNA levels of *GPX4* and *HO-1* in heart tissues of mice. The data were presented as mean ± SD, $^{*}P < 0.05$, $^{**}P < 0.01$, $^{***}P < 0.001$, ns, indicates no significance.

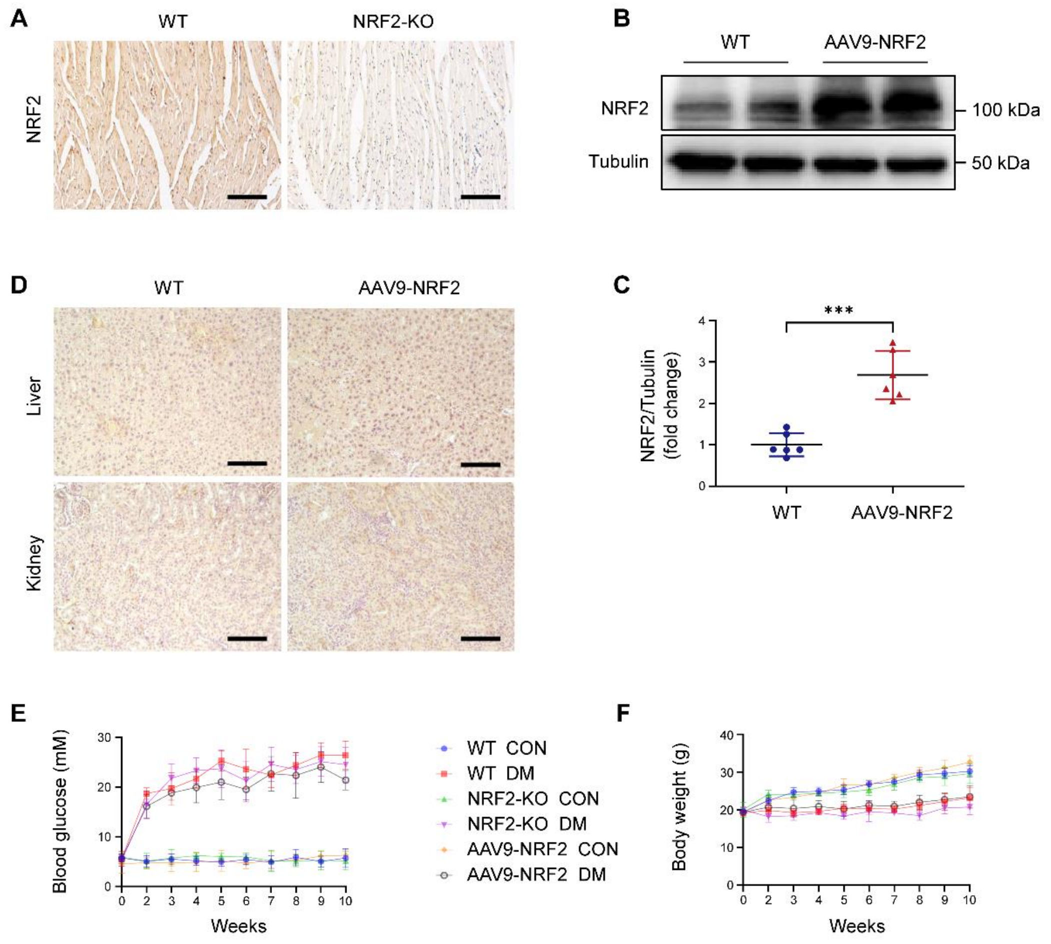

Figure S6. (**A**) Immunohistochemistry for NRF2 in heart tissues from WT or NRF2-KO mice. Scale bar = 200 μm. (**B-C**) Representative western blotting of NRF2 in heart samples from WT or AAV9-NRF2 mice. (**D**) Immunohistochemistry for NRF2 in liver and kidney from WT or AAV9-NRF2 mice. Scale bar = 100 μm. (**E**) The blood glucose was measured weekly. (**F**) The body weight was measured weekly. Data were expressed as mean ± SD. *** $P < 0.001$.

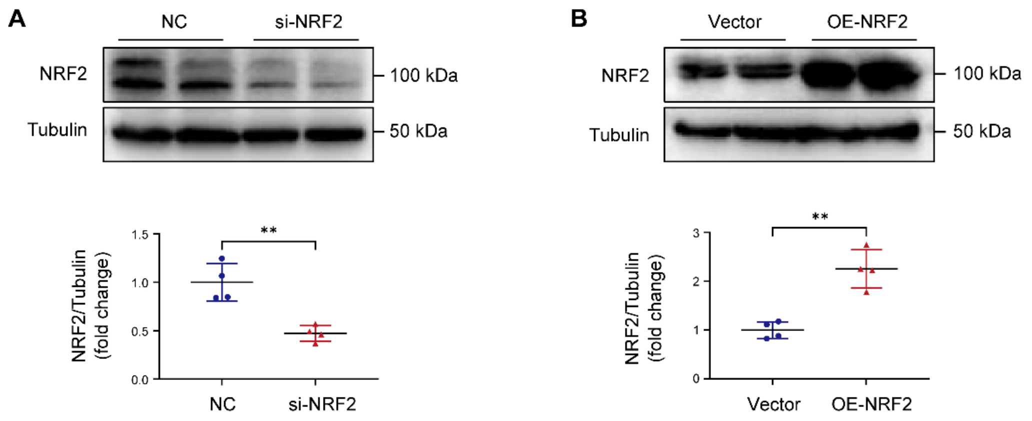

Figure S7. (**A**) Western blotting and quantification of NRF2 in NRVMs transfected with si-NC or si-NRF2 for 48 hours. (**B**) Western blotting and quantification of NRF2 in NRVMs infected with Lenti-Vector or Lenti-NRF2 for 48 hours. Data were expressed as mean ± SD. $^{**}P < 0.01$.

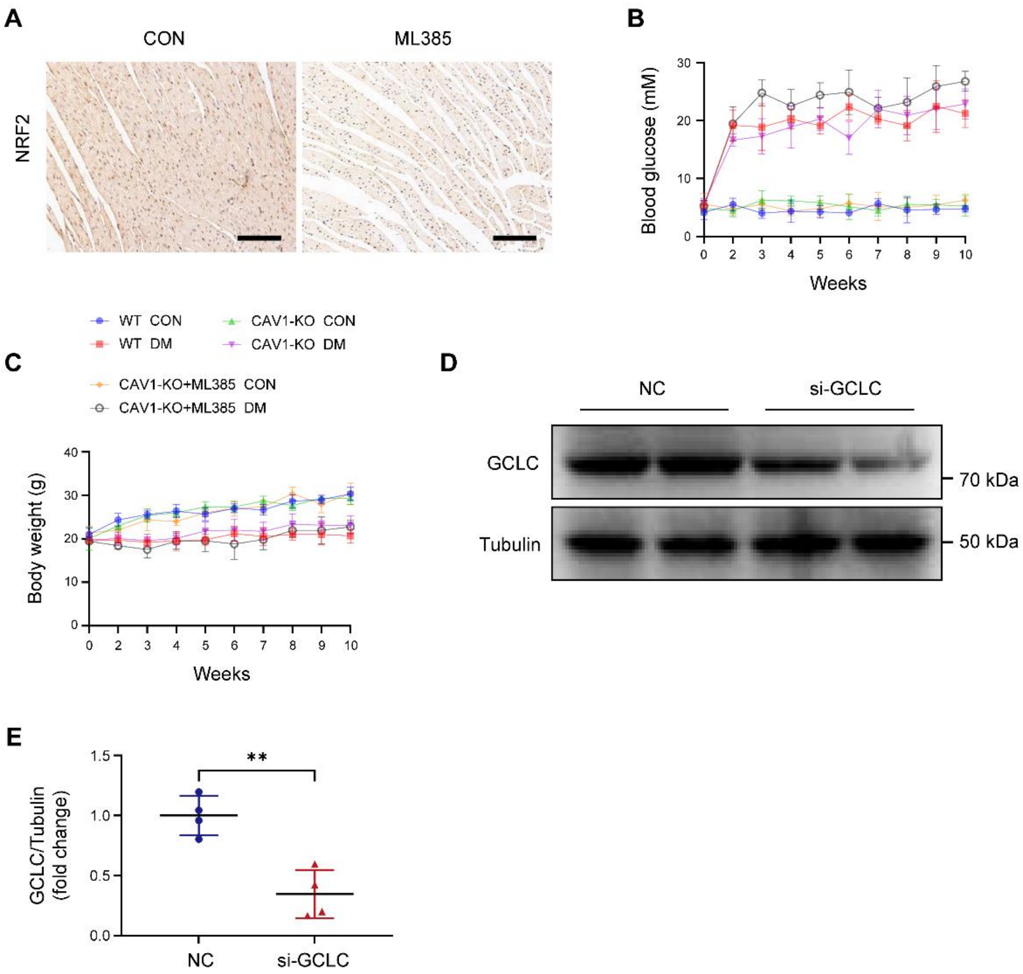

Figure S8. (**A**) Immunohistochemistry for NRF2 in heart sections. Scale bar = 200 μm. (**B**) The blood glucose was measured weekly. (**C**) The body weight was measured weekly. (**D-E**) Representative western blotting images and quantification of GCLC in NRVMs infected with si-NC or si-GCLC for 48 hours. Data were expressed as mean ± SD. $^{**}P < 0.01$.

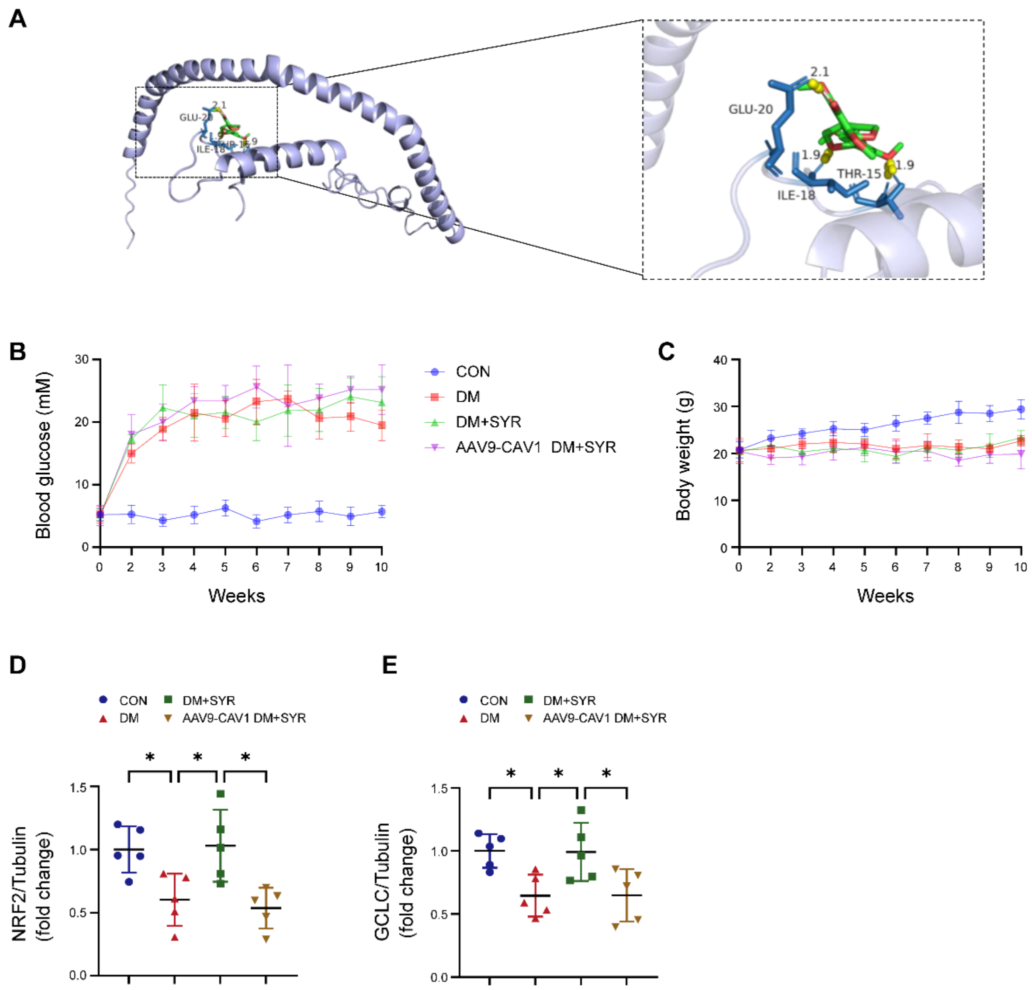

Figure S9. (**A**) Binding modes of SYR and CAV1 by molecular docking analysis. (**B**) The blood glucose was measured weekly. (**C**) The body weight was measured weekly. (**D-E**) Density quantification of representative western blots for NRF2 and GCLC. Representative western blotting images are shown in Figure 8N. Data were expressed as mean ± SD. $^{*}P < 0.05$, $^{**}P < 0.01$.
